# Early Initiation of Breastfeeding and Exclusive Breastfeeding in Anglophone and Francophone West African Countries: Systematic Review and Meta‐Analysis of Prevalence

**DOI:** 10.1111/mcn.13792

**Published:** 2025-01-07

**Authors:** Martha Osekua Lewis‐Koku, Catharine A. K. Fleming, Lucy Ngaihbanglovi Pachuau, Nagwa Farag Elmighrabi, Kingsley Emwinyore Agho

**Affiliations:** ^1^ School of Health Science Western Sydney University NSW Australia; ^2^ School of Health Science Western Sydney University Campbelltown NSW Australia; ^3^ Translational Health Research Institute (THRI) Western Sydney University Campbelltown NSW Australia; ^4^ Faculty of Health Sciences University of Johannesburg Johannesburg South Africa

**Keywords:** Anglophone, early initiation, exclusive breastfeeding, Francophone, indicators, prevalence, West Africa

## Abstract

Early initiation of breastfeeding (EIBF) and exclusive breastfeeding (EBF) are highly effective forms of preventive medicine in many low‐ and middle‐income countries, including Anglophone and Francophone West African countries. Despite the proven benefits of EIBF and EBF in reducing mortality and morbidity, there is limited systematic evidence from West African countries. Hence, the aim of this systematic review and meta‐analysis was to estimate the pooled prevalence of EIBF and EBF in Anglophone and Francophone West African countries. Six databases were searched for eligible studies based on inclusion criteria and a systematic review and a further meta‐analysis were done. The weighted prevalence of EIBF was 51.7% (95% CI: 48.8, 54.6) in Anglophone West African countries and 45.5% (95% CI: 42.0, 48.9) in Francophone West African countries. The pooled prevalence of EBF was 41.2% (95% CI: 36.9, 45.5) in Anglophone West African countries and 30.1% (95% CI: 26.7, 33.5) in Francophone West African countries. Our study showed that the weighted average EIBF and EBF prevalence tended to be higher from studies in Anglophone West African countries than in Francophone West African countries. Despite these findings, EIBF and EBF rates overall in West African countries were suboptimal. A substantial improvement is necessary in promoting EIBF and EBF in West African countries. Programmes should target all mothers in the region no matter their colonial allegiance to achieve Sustainable Development Goals 2 and 3 by 2030.

## Summary

- Breastfeeding practice is suboptimal and remains a key public health issue in Anglophone and Francophone West African countries.
- The prevalence of EIBF and EBF shows regional variation across West Africa, with weighted prevalence rates of EIBF at 51.7% in Anglophone and 45.5% in Francophone countries, and EBF at 41.2% and 30.1%, respectively.
- Intervention programmes should target all mothers in the region no matter their colonial allegiance to achieve sustainable development goals 2 and 3 by 2030.

## Graphical abstract

The weighted average prevalence of EIBF and EBF is higher in Anglophone countries than in Francophone countries in West Africa. However, both regions are suboptimal, and urgent intervention efforts should be context‐specific but target the whole subregion for progress in achieving SDG 2 and 3.

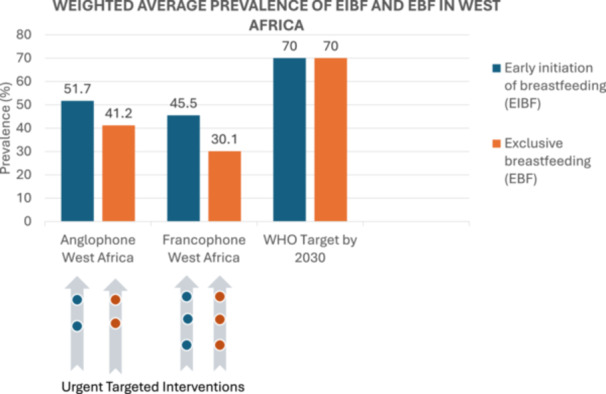

## 1 Introduction

Globally, 4.9 million children under five and 2.3 million neonates died in 2022 due to suboptimal breastfeeding (UNICEF 2024a, 2024b). Optimal breastfeeding practices, including early initiation and exclusive breastfeeding, improve infant nutrition and survival (World Health Organisation 2024). In 2023, the global rate of early initiation of breastfeeding (EIBF) was estimated at 46%, below the 70% target set for 2030. The rate varies from 39% in South Asia to 69% in Eastern and Southern Africa. Similarly, the exclusive breastfeeding (EBF) rate varied from 26% in North America to 60% in South Asia, with a global estimate of 48% which is close to the 50% target for 2025 but still falls short of the 70% target for 2030 (World Health Organisation 2023a).

The first 2000 days of a child's life are a crucial window for growth and development (Binns, Lee, and Low 2016; Micah projects 2023), during which EIBF and EBF play a vital role in reducing the risk of infectious and noninfectious diseases, reduce the risk of sudden infant death, improve cognitive ability, support favourable primary dentition (Binns, Lee, and Low 2016; Brahm and Valdes 2017; Salone, Vann, and Dee 2013) and are a more cost‐effective means to reduce the need for medical care and healthcare expenditure (Ball and Bennett 2001; Lechosa‐Muñiz et al. 2020). In particular, EIBF and EBF have been found to reduce the prevalence and duration of diarrhoea in children and its associated mortality among low‐ and middle‐income countries (LMIC), including West Africa (Hamer et al. 2022).

In West and Central Africa, the under‐five mortality rate (U5MR) ranges from 14 deaths per 1000 live births in Cape Verde to 115 in Niger, with an average of 91.5 deaths per 1000 live babies. The neonatal mortality rate (NMR) is 30.5 deaths per 1000 live births, the highest of all regions (UNICEF 2023b). Unfortunately, breastfeeding practices in the region are low, with EIBF at 39% and EBF at 38% (UNICEF 2023a). Most countries in West Africa are not on track to meet SDG breastfeeding targets (The Institute for Health Metrics and Evaluation 2020).

Multiple studies have been undertaken in West Africa on EIBF and EBF. From these, the prevalence of EIBF and EBF was found to be approximately 43% and 32.64%, respectively (Ameyaw et al. 2023; Appiah et al. 2021; Armah‐Ansah et al. 2023; Berde and Yalcin 2016; Boakye‐Yiadom et al. 2021; Ezeh et al. 2019; Issaka, Agho, and Renzaho 2017). Some of these studies focused only on specific countries, while others looked at broader regional categories such as the Economic Community of West African States (ECOWAS), sub‐Saharan Africa or LMIC status. Additionally, some studies examined specific subpopulations, such as women who delivered at health facilities. In certain studies, the timing of breastfeeding initiation was categorised (< 1, 1–6h, < 24 h, etc.). While pooled prevalence within sub‐Saharan Africa is important, it may not accurately reflect the situations and differences in Anglophone (English‐speaking) and Francophone (French‐speaking) West Africa, leading to potential inaccuracies in data used for local policies and decisions. A cross‐sectional study in the ECOWAS region, covering 13 out of the 15 countries in West Africa, also found a pooled prevalence of 43% for EIBF. However, this study did not fully account for the cultural and administrative differences between Anglophone and Francophone regions within West Africa as these regions vary by official national languages, education, income levels and Gross Domestic Product, religion and traditional practices at the birth of a baby which could impact feeding practices.

Currently, there are no studies specifically examining the prevalence of EIBF and EBF in the Anglophone and Francophone countries in West Africa independently. These countries differ in terms of colonial ties, official national language, culture, administrative systems, and legal and socio‐political systems (Akinyemi n.d.; Spotlight on Francophone Regions 2011) which may impact culture (Okazaki, David, and Abelmann 2008; Ziltener and Kunzler 2013). Evidence within these countries is necessary to establish the current prevalence of EIBF and EBF to evaluate progress towards achieving the sustainable development goals (SDGs). This will help determine the urgency and scale of targeted interventions required for more efficient and cost‐effective outcomes.

This study aims to fill this gap by conducting a systematic review and meta‐analysis to determine the prevalence of EIBF and EBF practices among women in Anglophone and Francophone countries in West Africa. The findings of the study are specific to these countries and may guide policymakers in tailoring breastfeeding programmes according to their cultural and administrative systems. Findings may contribute to more efficient and practical implementation of targeted breastfeeding interventions aimed at improving child nutrition, growth, and survival in West Africa, and ensure that the region remains on track to meet the SDGs by 2030.

## 2 Methods

The current study conducted a systematic review followed by a meta‐analysis of eligible studies. The Preferred Reporting Items for Systematic Reviews and Meta‐analyses (PRISMA) 2020 guidelines were used to review the existing literature. The PRISMA 2020 checklists for the manuscript and abstract are attached as Tables S1 and S2.

### 2.1 Study Area

West Africa is the westernmost part of Africa covering an area of 5,112,903 km^2^ with an estimated population of 418,544,337 (2021) and a total Gross Domestic Product (PPP) of US$2.091 trillion (2022) (West Africa 2024). The United Nations classifies 16 countries into the West African subregion (Wikipedia 2023). West Africa is categorised into two based on colonisation. Anglophones (English‐speaking) are British colonies and include The Gambia, Ghana, Liberia, Nigeria and Sierra Leone. Francophones (French‐Speaking) are French colonies comprising Benin, Burkina Faso, Cape Verde, Cote d'Ivoire, Guinea, Guinea Bissau, Mali, Mauritania, Niger, Senegal and Togo. For the purposes of this study, Mauritania will be excluded as it is usually associated with the Maghreb region and has closer ties with North African countries (Wikipedia 2023). Due to the influence of this compartmentalisation on relationships, ties fostered along colonial lines are much stronger than with neighbouring countries. Official national languages (and by extension culture) and administrative systems differ in these subsectors (Akinyemi). The Francophones share more than just a language but also share similar legal and socio‐political systems (Spotlight on Francophone Regions 2011).

### 2.2 Outcome Variables

The current systematic review employed two Infant and Young Child Feeding (IYCF) indicators defined below (World Health Organisation and The United Nations Children's Fund 2021):

1. EIBF: Percentage of children born in the last 24 months who were put to the breast within 1 h of birth.
2. EBF under 6 months (EBF): Percentage of infants 0–5 months of age who were fed exclusively with breast milk during the previous day.

### 2.3 Search Strategy

The systematic review was built on the following search protocol. Six databases (Ovid MEDLINE, Embase (Ovid), ProQuest, APA PsycINFO, CINAHL and Scopus) were scanned for relevant peer‐reviewed articles. Additionally, MeSH headings with related subheadings were combined with key terms for the included countries and regions. This was to ensure that all relevant studies were identified. Also, the reference lists of retrieved articles and Google Scholar were screened for further relevant publications. Articles retrieved were imported from each database into the EndNote library. The following combination of keywords was used in the search:

(Breastfeed* or “Breast feed*” or “Infant feed*” or Feed* or “Early Initiat*” or “Exclusive breast feed*” or “Breastfeeding Indic*”)

And

(Prevalence* or rate*)

And

(Gambia or Ghana or Liberia or Nigeria or “Sierra Leone” or Benin or “Burkina Faso” or “Cape Verde” or “Cote d'Ivoire” or Guinea or “Guinea Bissau” or Mali or Niger or Senegal or Togo or “Sub Sahara*” or ECOWAS or “West Africa*”).

### 2.4 Eligibility Criteria

Studies that met the following criteria were included in the review.

(i) Observational studies; (ii) Published in peer‐reviewed journals; (iii) Studies published in English between 1 January 2008 to 31 March 2024 (2008 was the inception of the World Health Organisation (WHO) breastfeeding indicators); (iv) Studies reporting on the prevalence of EIB and/or EBF; and (v) conducted on women with children in any or all of the West African countries (The Gambia, Ghana, Liberia, Nigeria, Sierra Leone, Benin, Burkina Faso, Cape Verde, Cote d'Ivoire, Guinea, Guinea Bissau, Mali, Niger, Senegal and Togo). Studies that were (i) Non‐peer‐reviewed articles, (ii) Reviews, (iii) Full text not accessible and (iv) not demonstrating a clear research methodology, for example, commentaries, editorials and letters and (v) HIV‐positive mothers were excluded. (World Health Organisation 2023b). The outcome measure of this study is the prevalence of EIBF and EBF.

### 2.5 Data Extraction

The articles identified in the search were exported into Endnote 20 (Clarivate Analytics, USA). Duplicated citations were removed, and screening and selection of the remaining articles were done. The first author (MOLK) screened the titles of the remaining publications. This was followed by reading the abstracts of studies retained from the first phase and eligible articles were retained for full‐text reading by MOLK. NFE, blinded to MOLK, assessed the article selection processes. At each stage, we checked the number of articles and discussed any differences of opinion. MOLK assessed the quality of the eligible studies and extracted the relevant data. Two authors LNP and NFE checked the relevance of the articles, and the details were recorded. All disagreements between the reviewers were resolved through discussion and a consensus on potential eligibility was reached. Another superior reviewer KEA was available to resolve differences that emerged. These were to ensure the reliability of the study. The data were then classified into EIBF‐ or EBF‐related and Anglophone or Francophone depending on the country in which the study was done. The summary of the selected studies is recorded in Table S7, and this includes author, year of publication, study setting, country category, population characteristics, sample strategy, study design and quality assessment score.

### 2.6 Quality Assessment

The JBI critical appraisal tool was used to evaluate the degree of reliability, validity and usefulness of the identified studies and to assess the quality of the reviewed studies. The tool comprises a checklist of nine questions used to evaluate the external validity (bias in selection) and internal validity (information biases and bias of cofounders) (Munn et al. 2015). The accuracy, suitability and confidentiality of the JBI tool for critical appraisal of systematic reviews were confirmed (Munn et al. 2020). For each reviewed study, a score ranging from zero to nine was allotted (zero if none of the criteria were met, and nine if all the criteria were met). The total points allotted determined the overall quality of the study. Studies were rated as being high quality (7–9), medium (4–6) or poor quality (0–3), as shown in Table S3.

### 2.7 Statistical Methods

All results with complete variables of interest were imported into STATA version 18 (Stata Corp LLC, Texas, USA). For each country, the prevalence of EIBF and EBF was assessed through the forest plot. A forest plot is necessary to synthesise data in an observational study. The forest plots with their corresponding weight, 95% confidence interval and weighted prevalence in the Anglophone and Francophone countries were displayed for each indicator. A high level of inconsistency (*I* ^2^ > 50%) was observed in a heterogeneity test indicating the use of a random effect model in the meta‐analysis conducted. Subsequently, a funnel plot and Begg's test were conducted to visually detect the presence of publication bias. The interactions between Anglophone and Francophone and the year of publication for both EBF and EIBF were assessed using ‘metapreg’ command in STATA. The likelihood ratio test statistic was applied to test the significant difference between the weighted average prevalence of EIBF and EBF of the two groups (Anglophone and Francophone) West African countries and the year of publication.

## 3 Results

**Figure 1:**
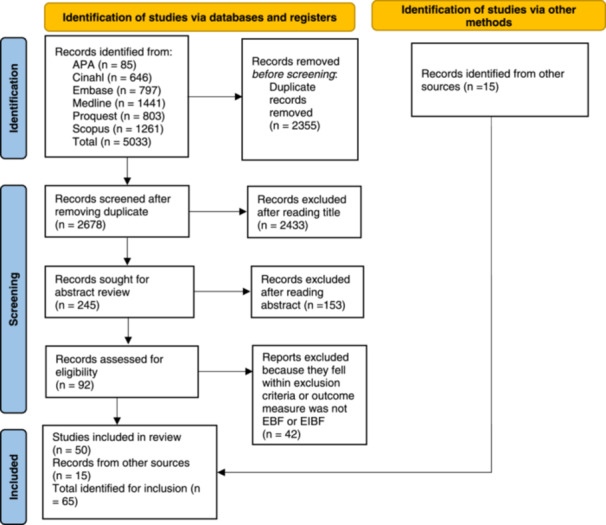
Flow chart of the study selection process. PRISMA 2020 flow diagram.

A total of 5033 peer‐reviewed articles were initially identified from the six databases as shown in Figure 1. After removing 2355 duplicates, 2678 articles progressed to the next phase. Screening of titles excluded 2433 articles, and screening of abstracts excluded a further 153. Full text of the remaining 92 was screened, and 38 were excluded based on inclusion and exclusion criteria leaving 54 eligible articles. A further search identified 15 papers from Google Scholar and the reference list of eligible articles. Hence, 69 peer‐reviewed papers were included in the studies for critical appraisal.

### 3.1 Characteristics of Studies Included

All 69 studies included in this review were cross‐sectional and represented all 15 countries in West Africa. Further details about the included studies can be found in Table S7. Forty‐two (60.9%) of the included studies investigated EIBF; 26 were studies done in Anglophone, 4 were studies done in Francophone and 12 were multi‐country studies covering both Anglophone and Francophone. Forty‐six (66.7%) of the included studies investigated EBF; 33 were studies done in Anglophone, 6 were studies done in Francophone and 7 were multi‐country studies covering both Anglophone and Francophone countries. Again, 19 (27.5%) studies investigated EIBF and EBF. Most studies used data from nationally representative surveys except for 27 that used primary data sources. The age range of the study samples is within the 15‐ to 49‐year reproductive period who have children 0–24 months. On assessing the quality of the studies included using the JBI criteria, 64 (92.8%) were scored high and 5 (7.2%) were scored medium. No study was scored low as seen in Table S3. Our study involved a total of 1,032,003 reported participants with the sample size of included studies ranging from 36 to 150,312.

### 3.2 Meta‐Analysis of Prevalence

Of the 69 studies that were reviewed, 4 were excluded from the meta‐analysis due to non‐reporting of sample size (N or n) (Bergamaschi, Oakley, and Benova 2019; Ekholuenetale et al. 2022; Oakley et al. 2018; Pretorius et al. 2021).

#### 3.2.1 Early Initiation of Breastfeeding

**Figure 2:**
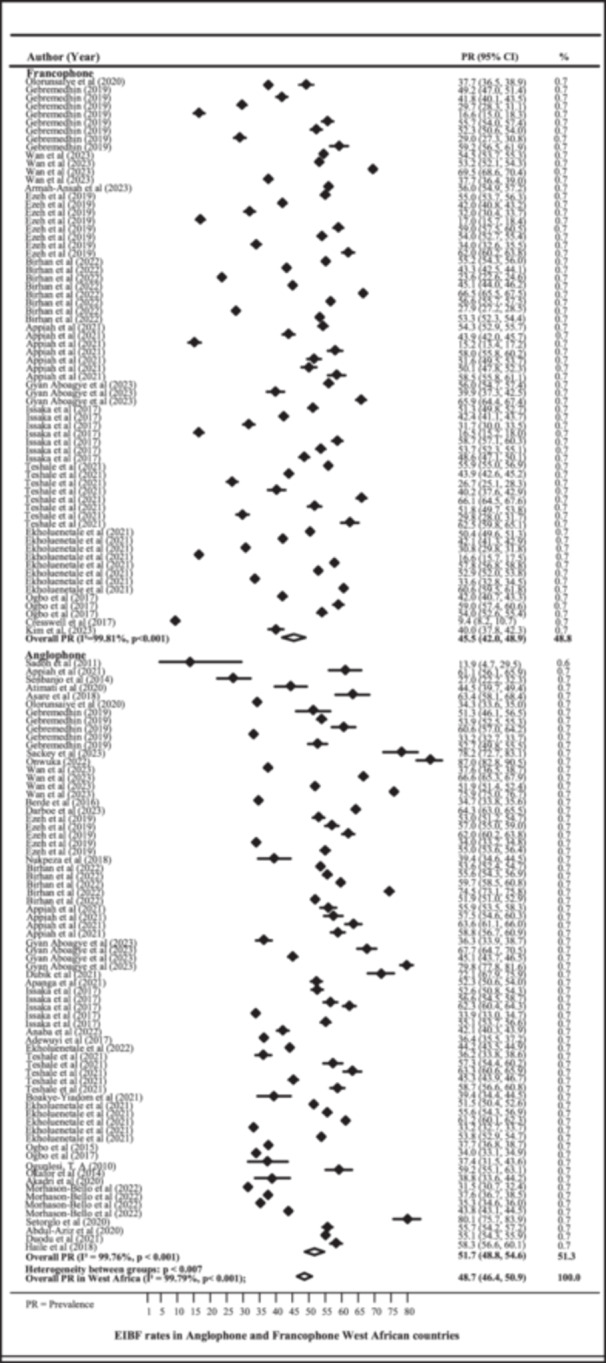
Prevalence of EIBF in Anglophone and Francophone West African countries.

Figure 2 shows a forest plot of the prevalence of EIBF among Anglophone and Francophone West African countries. The prevalence of EIBF in Anglophone West African countries was 51.7% (95% CI 48.8, 54.6) with a heterogeneity of 99.76%. The prevalence of EIBF in Francophone West African countries was 45.5% (95% CI 42.0, 48.9), with a heterogeneity of 99.81%. The overall random effect weighted prevalence of EIBF for the whole West African geographic zone was approximately 48.7% (95% CI 46.4, 50.9) with heterogeneity (*I*² = 99.79%). High heterogeneity (> 50%) indicates that variability in the included studies is most likely due to diversity between the studies themselves and not due to chance. The Likelihood Ratio (LR) test statistic for the interaction of subregions (both anglophones Vs francophone) by year of publication revealed that the prevalence of EIBF is unequal (χ^2^ = 495.18, *p*‐value < 0.001). In studies comparing EIBF in Anglophone and Francophone West African countries, it was found that there was consistency in the results (homogeneity) using funnel plots and Begg's test (Supporting Information S1: Figure 2). Additionally, a meta‐regression analysis showed that the proportion of EIBF increased over the years of publication, as shown in Supporting Information S1: Figure 4.

#### 3.2.2 Exclusive Breastfeeding

**Figure 3:**
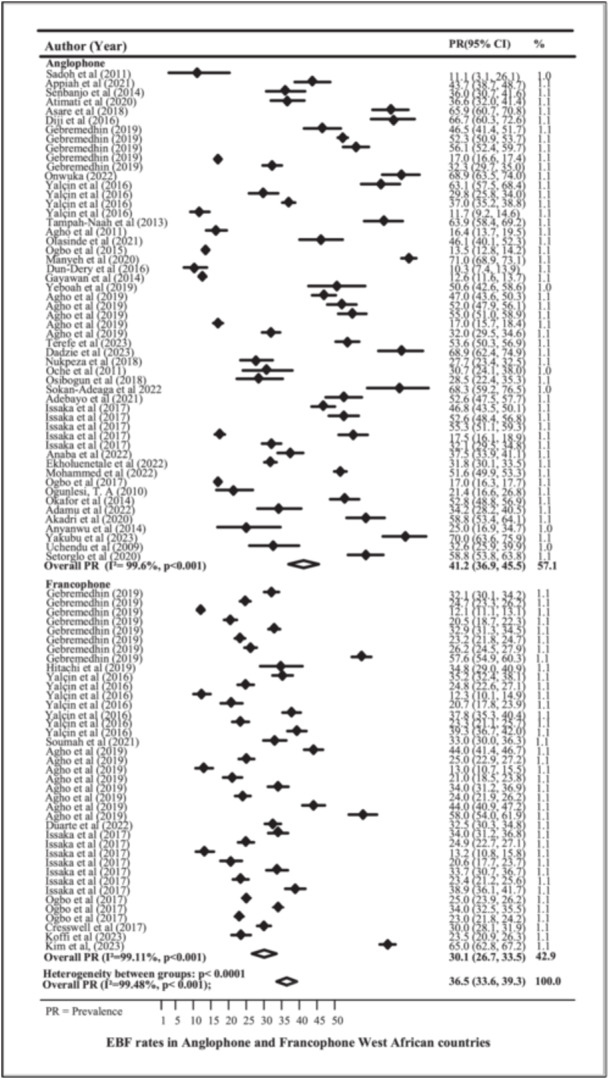
Prevalence of EBF in Anglophone and Francophone West African countries.

Figure 3 shows a forest plot of the prevalence of EBF among Anglophone and Francophone West African countries. The prevalence of EBF in Anglophone West African countries was 41.2% (95% CI 36.9, 45.5), with a heterogeneity of 99.6%. The prevalence of EBF in Francophone West African countries was 30.1% (95% CI 26.7, 33.5) with a heterogeneity of 99.11%. The overall random effect weighted prevalence of EBF for the whole West African geographic zone was approximately 36.5% (95% CI 33.6, 39.3) with heterogeneity (*I*² = 99.5%). High heterogeneity (> 50%) indicates that variability in the included studies is most likely due to diversity between the studies themselves and not due to chance. Funnel plots were used, and Begg's test was conducted to analyse EBF rates in Anglophone and Francophone West African countries. The LR test statistic for the interaction of subregions (both anglophones vs francophones) by year indicates that the prevalence of EBF varies (*χ* ^2^ = 381.41, *p*‐value < 0.001). The results indicated that there was no publication bias (see Supporting Information S1: Figure 1). Additionally, meta‐regression analysis was performed to assess the relationship between EBF and the year of publication, revealing that the proportion of EBF increased with each subsequent year of publication, as illustrated in Supporting Information S1: Figure 3.

## 4 Discussion

The current study investigated the prevalence of EIBF and EBF practices among women in Anglophone and Francophone countries in West Africa. In this study, 69 studies published within and after the year of introduction of the IYCF indicators met the eligibility criteria to determine the prevalence of EIBF and EBF among this population. Most (98.2%) of the studies included in the review were rated as high in quality and had adequate representation of sample size and country coverage. Hence, the outcome evidence may be considered highly reliable, valid and useful. The study further categorised the 15 West African countries into Anglophone and Francophone and carried out a meta‐analysis to examine the pooled prevalence of EIBF and EBF. We found that the weighted prevalence of EIBF and EBF rates were suboptimal but higher in Anglophone than Francophone West African countries. These differences in EIBF and EBF rates did not differ statistically because their 95% confidence intervals overlapped. The proportion of EIBF and EBF increased with each subsequent year of publication. The EIBF and EBF rates suggest the urgent need for breastfeeding promotion especially among mothers who live in Francophone West African countries.

Our study showed that EIBF and EBF practices in both Anglophone and Francophone West African countries were suboptimal and fell short of the 70% target for 2030. In absolute figures, our findings for EIBF were lower in Anglophones and much lower in Francophones than the 57.6% average reported in the WHO Global survey (Takahashi et al. 2017) but consistent with a study involving 57 LMIC, including West African countries which indicated suboptimal levels of EIBF (51.9%) and EBF (45.7%) (Wu et al. 2021). Similarly, EBF for Francophones was also much lower than the 43% reported in another cross‐sectional study among African children (Ekholuenetale et al. 2022). A systematic review of the prevalence of breastfeeding (BF) in industrialised economies revealed a poor breastfeeding culture in the United Kingdom and France compared to Scandinavia and Japan (Ibanez et al. 2012). Britain and France may have passed on the poor BF culture to their colonies. Additionally, inequitable distribution of healthcare facilities and high rural residence may have contributed to the observed prevalences (Agho et al. 2019; Ezeh et al. 2019). A scale‐up of BF intervention strategies is needed in the whole West African region.

The current research reveals a positive association between the year of publication and the prevalence of EIBF and EBF, which may be explained by the increasing implementation of breastfeeding interventions over time, such as IYCF indicators and the Baby‐Friendly Hospital Initiative (BFHI). With a varied uptake, however, increased exposure to the BFHI 10 Steps demonstrably contributes to improved short‐, medium‐ and long‐term breastfeeding outcomes including the initiation of breastfeeding during the postpartum period and establishment of EBF (Pérez‐Escamilla, Martinez, and Segura‐Pérez 2016; World Health Organisation and The United Nations Children's Fund 2021). Unfortunately, only about 60% of deliveries in Sub‐Saharan Africa (SSA) occur in health facilities (Gage et al. 2021) and less than 4% of deliveries in Africa occur in BFHI‐designated hospitals (World Health Organisation 2017). The poor hospital delivery implies less access to maternal and essential newborn care practices including EIBF and EBF, and may reflect the slow U5MR (168 to 91) and NMR (43 to 31) decline within the West and Central African Region, from 2000 to 2021 (UNICEF 2023b). Studies have revealed that EIBF and EBF, in particular, reduce the prevalence, severity and duration of diarrhoea in children and its associated mortality among LMIC, including in West Africa (Hamer et al. 2022; Prentice 2022). Scaling up breastfeeding practice interventions may improve EIBF and EBF prevalence and child survival in countries like West African countries.

Our study also showed that the proportions of each indicator practised varied widely between and within Anglophone and Francophone countries in West Africa. In Anglophone West African countries, women exhibited a 6% higher likelihood of initiating breastfeeding within the first hour postpartum and an 11% greater probability of EBF for the initial 6 months compared to their counterparts in Francophone countries. The current evidence is consistent with the findings of a very large cross‐sectional study involving 29 African countries (Issaka, Agho and Renzaho 2017) indicating a difference in EIBF between Central Africa (38%) and Southern Africa (69%), and a nationally representative cross‐sectional study in India that showed an 11% difference margin in EBF between Southern India and North East India (Ogbo et al. 2019). This difference suggests the possibility of varied uptake levels of breastfeeding (BF) practices even among countries of similar geographies. Within similar geographies, a possible factor, colonialism, may be responsible for differences in prevalence. Our findings may imply that the low prevalence of EIBF and EBF in West Africa is largely influenced negatively by the Francophone countries whose colonial master, France, is ranked among the least likely to BF in Europe (Women Across Frontiers 2016) and lags behind The UK (Thompson 2024). Hence, population‐specific interventions may be plausible and more cost‐effective.

The findings observed from the study are strong as evidenced by the likelihood ratio test. The findings suggest a significant interaction between the subregion (Anglophones and Francophones) and the year of publication, meaning that the weighted average prevalence of EIBF differs significantly across these groups and years. The *χ* ^2^ statistic is very large (495.18), and the *p*‐value is extremely small (< 0.001). Similarly, the weighted average prevalence of EBF differs significantly across these groups and years. The chi‐square statistic is very large (381.41), and the *p*‐value is extremely small (< 0.001), further indicating that these differences are statistically significant and unlikely to be due to chance.

One major variation in EIBF and EBF prevalence in Anglophone and Francophone countries may be the effect of colonialism. Relatively similar neighbouring countries, Ghana (Anglophone) and Cote d'Ivoire (Francophone), have shown different healthcare trajectories due to colonialism (Vrooman 2023). Public investments in the colonial era focused less on the education and health sectors in West Africa. A study on colonial public investment in health and education in West and East Africa showed that Anglophones weaned off the colonial style of resource allocation quicker compared to the Francophones and increased investment in education and health (Ricart‐Huguet 2021) resulting in potential differences in educational levels, income levels and economic growth. Modifiable factors such as large rural residence (80% of population) with a lack of adequate standard French medium primary education in Burkina Faso (Trudell 2012), inadequate access to skilled professional delivery in Niger (Issoufou Kapran et al. 2023), inequitable distribution of healthcare facilities (Falchetta, Hammad, and Shayegh 2020) and religious and traditional practices (Sosseh, Barrow, and Lu 2023) may interfere with optimal breastfeeding practices resulting in low EIBF and EBF prevalences to varied extents. Improvement in BF prevalence may require interventions aligning with these modifiable factors.

Creating a supportive environment along social, cultural and political lines, in line with the WHO framework, is a key public health intervention to encourage acceptance and ownership of interventions (World Health Organisation 2009). Additionally, supportive interventions such as education and counselling are suitable strategies for promoting breastfeeding (Yas et al. 2023). It is critical for policymakers to focus urgently on scaling up the prevalence of EIBF and EBF practices in the Anglophone and Francophone countries in West Africa while placing greater emphasis on the Francophone countries. Francophones form about 67% of the region's representation. Hence, any effective intervention could potentially scale up prevalence and progress estimates within West Africa.

### 4.1 Strengths and Limitations

This study is limited by recall bias from the mothers, ignored variability of maternal populations and information bias due to many studies being conducted in Anglophone West African countries such as Nigeria and Ghana. The study is also limited by the fact that it primarily focused on articles published in English, excluding those written in French. This may lead to either an overestimation or underestimation of prevalence in Francophone countries. Despite these limitations, this study has several strengths: (1) Findings from the study can be generalised by increasing the strength of evidence relating to Anglophone and Francophone West African countries; (2) increases the statistical power of small studies; (3) reduces biases, such as publication bias; (4) identifies sources of diversity across various types of observational study design; and (4) reduces deficiencies in research study design, analysis and interpretations (Ioannidis and Lau 1999).

### 4.2 Policy Implications

This study provides much‐needed current evidence of EIBF and EBF prevalence in Anglophone and Francophone countries in West Africa. It serves as a needs assessment measure for the two indicators reporting low prevalence in the Anglophone and Francophone West African countries. Globally, it may help to determine progress towards achieving SDGs 2 and 3 while highlighting the difference in progress made in the Anglophone and Francophone countries. Governments, health authorities and programme managers may find it useful in formulating new and modified breastfeeding policies and intervention implementation strategies for better outcomes. These may be Anglophone and Francophone country‐specific and in line with their administrative, legal and socio‐political systems. Additionally, it may warrant increased research in the underrepresented countries to generate more data for informed decision‐making. Promoting EIBF and EBF practices can enhance breastfeeding practices to reduce undernutrition, stunted growth, morbidity and mortality among neonates and children under 5.

## 5 Conclusion

Suboptimal breastfeeding practice remains a problem in West Africa. The weighted average EIBF and EBF prevalence tended to be higher from studies in Anglophone West African countries than in Francophone West African countries, but both indicators fall short of global targets. Stakeholders in these subsectors will need to upscale their intervention efforts with a targeted approach to risk achieving the SDG targets by 2030. We recommend further studies to understand the factors associated with EIBF and EBF within Anglophone and Francophone West African countries. Additionally, more primary studies are needed in the countries underrepresented.

## Author Contributions

Conceptualisation: Martha Osekua Lewis‐Koku, Lucy Ngaihbanglovi Pachuau, Nagwa Farag Elmighrabi, Kingsley Emwinyore Agho. Data curation: Martha Osekua Lewis‐Koku. Formal analysis: Martha Osekua Lewis‐Koku. Investigation: Martha Osekua Lewis‐Koku. Methodology: Martha Osekua Lewis‐Koku, Lucy Ngaihbanglovi Pachuau, Nagwa Farag Elmighrabi, Kingsley Emwinyore Agho. Project administration: Martha Osekua Lewis‐Koku. Resources: Martha Osekua Lewis‐Koku. Software: Martha Osekua Lewis‐Koku, Kingsley Emwinyore Agho. Supervision: Kingsley Emwinyore Agho, Catharine A. K. Fleming. Validation: Martha Osekua Lewis‐Koku. Visualisation: Martha Osekua Lewis‐Koku. Writing– original draft: Martha Osekua Lewis‐Koku. Writing– review & editing: Martha Osekua Lewis‐Koku, Catharine A. K. Fleming, Lucy Ngaihbanglovi Pachuau, Nagwa Farag Elmighrabi, Kingsley Emwinyore Agho. All authors have read and approved the final manuscript.

## Conflicts of Interest

The authors declare no conflicts of interest.

## Supporting information

S1 Fig. Funnel plots and 95% confidence intervals of EBF.
S2 Fig. Funnel plots and 95% confidence intervals of EIBF.
S3 Fig. Meta‐regression analysis of EBF
S4 Fig. Meta‐regression analysis of EIBF.

S1 Table. PRISMA 2020 checklist for the main.

S2 Table. PRISMA 2020 checklist for the abstract.

S3 Table. Quality assessment score.

S4 Table. Prevalence of EIBF among Anglophone and Francophone West African countries.

S5 Table. Prevalence of EBF among Anglophone and Francophone West African countries.

S6 Table. Selection strategy for one database.

S7 Table. Characteristics of included studies.

## Data Availability

Data sharing is not applicable to this article as no new data were created or analysed in this study. The sample size was not always explicitly stated in studies. The authors, however, direct readers to the name of the national data report or a link for more information. Demographic Health Survey (DHS) reports and databases for each country are available @ dhsprogram.com; information about Multiple Indicator Cluster Surveys (MIC) is available @ mics. unicef.org.
