# Supplementary material for: Early Initiation of Breastfeeding and Exclusive Breastfeeding in Anglophone and Francophone West African Countries: Systematic Review and Meta‐Analysis of Prevalence
Source: Matern Child Nutr. 2025 Jan 7;21(2):e13792. doi: 10.1111/mcn.13792 (PMC11956053; doi:10.1111/mcn.13792)
Supplement: Supplementary file 1 — S1 Fig. Funnel plots and 95% confidence intervals of EBF. S2 Fig. Funnel plots and 95% confidence intervals of EIBF. S3 Fig. Meta‐regression analysis of EBF S4 Fig. Meta‐regression analysis of EIBF. [file MCN-21-e13792-s007.docx]

S1 Fig: Funnel plot with 95%CI for Exclusive Breastfeeding by Anglophone and Francophone West African countries

S2 Fig: Funnel plot with 95%CI for Early Initiation of Breastfeeding (EIBF) by Anglophone and Francophone West African countries

S3Fig showed a meta-regression analysis of EBF by year of publication. The vertical axis is the log proportion of EBF, and the horizontal axis represents the year of publication. Each dark dot represented one selected study, and the size of each dark dot corresponds to the weight assigned to each study. Given the slope of the regression line has descended slightly in this figure, this could be interpreted as the publication of the year increased, the proportion of EBF increased, and this relationship differed statistically (p < 0.001)

S3 Fig: Meta-regression analysis of EBF

S4 Fig showed a meta-regression analysis of EIBF by year of publication. The vertical axis is the log proportion of EIBF, and the horizontal axis represents the year of publication. Each dark dot represented one selected study, and the size of each dark dot corresponds to the weight assigned to each study. Given the slope of the regression line has descended slightly in this figure, this could be interpreted as the publication of the year increased, the proportion of EIBF increased, and this relationship differed statistically (p < 0.001)

S4 Fig: Meta-regression analysis of EIBF
