# Supplementary material for: Early Initiation of Breastfeeding and Exclusive Breastfeeding in Anglophone and Francophone West African Countries: Systematic Review and Meta‐Analysis of Prevalence
Source: Matern Child Nutr. 2025 Jan 7;21(2):e13792. doi: 10.1111/mcn.13792 (PMC11956053; doi:10.1111/mcn.13792)
Supplement: Supplementary file 4 — S3 Table. Quality assessment score. [file MCN-21-e13792-s005.docx]

**Table 2: Quality Assessment score for studies included in the review of EIBF and EBF**

| **Author (Year)** | **1. Was the sample frame appropriate to address the target population?** | **2.         Were study participants sampled in an appropriate way?** | **3.         Was the sample size adequate?** | **4.         Were the study subjects and the setting described in detail?** | **5.         Was the data analysis conducted with sufficient coverage of the identified sample?** | **6.         Were valid methods used for the identification of the condition?** | **7.         Was the condition measured in a standard, reliable way for all participants?** | **8.         Was there appropriate statistical analysis?** | **9.       Was the response rate adequate, and if not, was the low response rate managed appropriately?** | **Quality assessment score** |
| --- | --- | --- | --- | --- | --- | --- | --- | --- | --- | --- |
| Abdul-Aziz et al (2020) | 1 | 1 | 1 | 1 | 1 | 1 | 1 | 0 | 1 | 8 |
| Adamu et al (2022) | 1 | 1 | 1 | 1 | 1 | 1 | 1 | 0 | 1 | 8 |
| Adebayo et al (2021) | 1 | 1 | 0 | 1 | 1 | 1 | 1 | 1 | 1 | 8 |
| Adewuyi et al (2017) | 1 | 1 | 1 | 1 | 1 | 1 | 0 | 1 | 1 | 8 |
| Agho et al (2011) | 1 | 1 | 1 | 1 | 1 | 1 | 1 | 1 | 1 | 9 |
| Agho et al (2019) | 1 | 1 | 1 | 1 | 1 | 1 | 1 | 1 | 1 | 9 |
| Akadri et al (2020) | 1 | 1 | 1 | 1 | 1 | 1 | 0 | 0 | 1 | 7 |
| Anaba et al (2022) | 1 | 1 | 1 | 1 | 1 | 1 | 1 | 1 | 1 | 9 |
| Anyanwu et al (2014) | 1 | 1 | 0 | 1 | 1 | 1 | 0 | 0 | 1 | 6 |
| Apanga et al (2021) | 1 | 1 | 1 | 1 | 1 | 1 | 1 | 1 | 1 | 9 |
| Appiah et al (2021) | 1 | 1 | 1 | 1 | 1 | 1 | 1 | 0 | 1 | 8 |
| Appiah et al (2021) | 1 | 1 | 1 | 1 | 1 | 1 | 1 | 0 | 1 | 8 |
| Armah-Ansah et al (2023) | 1 | 1 | 1 | 1 | 1 | 1 | 1 | 1 | 1 | 9 |
| Asare et al (2018) | 1 | 1 | 1 | 1 | 1 | 1 | 1 | 1 | 1 | 9 |
| Atimati et al (2020) | 1 | 1 | 0 | 1 | 1 | 1 | 0 | 1 | 1 | 7 |
| Berde et al (2016) | 1 | 1 | 1 | 1 | 1 | 1 | 1 | 1 | 1 | 9 |
| Bergamaschi et al (2019) | 1 | 1 | 0 | 1 | 1 | 1 | 1 | 1 | 0 | 7 |
| Birhan et al (2022) | 1 | 1 | 1 | 1 | 1 | 1 | 1 | 1 | 1 | 9 |
| Boakye-Yiadom et al (2021) | 1 | 1 | 1 | 1 | 1 | 1 | 1 | 1 | 1 | 9 |
| Cresswell et al (2017) | 1 | 1 | 1 | 1 | 1 | 1 | 1 | 0 | 1 | 8 |
| Dadzie et al (2023) | 1 | 1 | 1 | 1 | 1 | 1 | 1 | 0 | 1 | 8 |
| Darboe et al (2023) | 1 | 1 | 1 | 0 | 1 | 1 | 1 | 0 | 1 | 7 |
| Diji et al (2016) | 1 | 1 | 1 | 1 | 1 | 1 | 0 | 0 | 1 | 7 |
| Duarte et al (2022) | 1 | 1 | 1 | 1 | 1 | 1 | 1 | 0 | 1 | 8 |
| Dubik et al (2021) | 1 | 1 | 1 | 1 | 1 | 1 | 1 | 0 | 1 | 8 |
| Dun-Dery et al (2016) | 1 | 1 | 1 | 1 | 1 | 1 | 1 | 0 | 1 | 8 |
| Duodu et al (2021) | 1 | 1 | 1 | 1 | 1 | 1 | 1 | 0 | 1 | 8 |
| Ekholuenetale et al (2021) | 1 | 1 | 1 | 1 | 1 | 1 | 1 | 1 | 1 | 9 |
| Ekholuenetale et al (2022) | 1 | 1 | 1 | 1 | 1 | 1 | 1 | 1 | 1 | 9 |
| Ekholuenetale et al (2022) | 1 | 1 | 0 | 1 | 1 | 1 | 1 | 1 | 0 | 7 |
| Ezeh et al (2019) | 1 | 1 | 1 | 1 | 1 | 1 | 1 | 1 | 1 | 9 |
| Gayawan et al (2014) | 1 | 1 | 1 | 1 | 1 | 1 | 1 | 0 | 1 | 8 |
| Gebremedhin (2019) | 1 | 1 | 1 | 1 | 1 | 1 | 1 | 0 | 1 | 8 |
| Gyan Aboagye et al (2023) | 1 | 1 | 1 | 1 | 1 | 1 | 1 | 1 | 1 | 9 |
| Haile et al (2018) | 1 | 1 | 1 | 1 | 1 | 1 | 1 | 0 | 1 | 8 |
| Hitachi et al (2019) | 1 | 1 | 1 | 1 | 1 | 1 | 1 | 0 | 1 | 8 |
| Issaka et al (2017) | 1 | 1 | 1 | 1 | 1 | 1 | 1 | 1 | 1 | 9 |
| Kim et al, (2023) | 1 | 1 | 1 | 1 | 1 | 1 | 1 | 0 | 1 | 8 |
| Koffi et al (2023) | 1 | 1 | 1 | 1 | 1 | 1 | 1 | 0 | 1 | 8 |
| Manyeh et al (2020) | 1 | 1 | 1 | 1 | 1 | 1 | 1 | 0 | 1 | 8 |
| Mohammed et al (2022) | 1 | 1 | 1 | 1 | 1 | 1 | 1 | 0 | 1 | 8 |
| Morhason-Bello et al (2022) | 1 | 1 | 1 | 1 | 1 | 1 | 1 | 1 | 1 | 9 |
| Nukpeza et al (2018) | 1 | 1 | 1 | 1 | 1 | 1 | 1 | 0 | 1 | 8 |
| Oakley et al (2018) | 1 | 1 | 0 | 1 | 1 | 1 | 1 | 1 | 0 | 7 |
| Oche et al (2011) | 1 | 1 | 1 | 1 | 1 | 1 | 1 | 0 | 1 | 8 |
| Ogbo et al (2015) | 1 | 1 | 1 | 1 | 1 | 1 | 1 | 1 | 1 | 9 |
| Ogbo et al (2017) | 1 | 1 | 1 | 1 | 1 | 1 | 1 | 0 | 1 | 8 |
| Ogunlesi, T. A (2010) | 1 | 0 | 0 | 1 | 1 | 1 | 0 | 0 | 1 | 5 |
| Okafor et al (2014) | 1 | 1 | 1 | 1 | 1 | 1 | 1 | 0 | 1 | 8 |
| Olasinde et al (2021) | 1 | 1 | 1 | 1 | 1 | 1 | 1 | 0 | 1 | 8 |
| Olorunsaiye et al (2020) | 1 | 1 | 1 | 1 | 1 | 1 | 1 | 0 | 1 | 8 |
| Olorunsaiye et al (2020) | 1 | 1 | 1 | 0 | 1 | 1 | 1 | 0 | 1 | 7 |
| Onwuka (2022) | 1 | 1 | 1 | 1 | 1 | 1 | 1 | 0 | 1 | 8 |
| Osibogun et al (2018) | 1 | 1 | 1 | 1 | 1 | 1 | 1 | 0 | 1 | 8 |
| Pretorius et al (2021) | 1 | 1 | 0 | 0 | 1 | 1 | 1 | 1 | 0 | 6 |
| Sackey et al (2023) | 1 | 1 | 1 | 1 | 1 | 1 | 1 | 0 | 1 | 8 |
| Sadoh et al (2011) | 1 | 0 | 0 | 1 | 1 | 1 | 0 | 0 | 0 | 4 |
| Senbanjo et al (2014) | 1 | 1 | 0 | 1 | 1 | 1 | 1 | 0 | 1 | 7 |
| Setorglo et al (2020) | 1 | 1 | 1 | 1 | 1 | 1 | 1 | 0 | 1 | 8 |
| Sokan-Adeaga et al 2022 | 1 | 1 | 1 | 1 | 1 | 1 | 1 | 0 | 1 | 8 |
| Soumah et al (2021) | 1 | 1 | 1 | 1 | 1 | 1 | 1 | 1 | 1 | 9 |
| Tampah-Naah et al (2013) | 1 | 1 | 1 | 1 | 1 | 1 | 1 | 0 | 1 | 8 |
| Terefe et al (2023) | 1 | 1 | 1 | 1 | 1 | 1 | 1 | 0 | 1 | 8 |
| Teshale et al (2021) | 1 | 1 | 1 | 1 | 1 | 1 | 1 | 1 | 1 | 9 |
| Uchendu et al (2009) | 1 | 1 | 0 | 1 | 1 | 1 | 0 | 0 | 1 | 6 |
| Wan et al (2023) | 1 | 1 | 1 | 1 | 1 | 1 | 1 | 1 | 1 | 9 |
| Yakubu et al (2023) | 1 | 1 | 1 | 1 | 1 | 1 | 1 | 0 | 1 | 8 |
| Yalçin et al (2016) | 1 | 1 | 1 | 1 | 1 | 1 | 1 | 0 | 1 | 8 |
| Yeboah et al (2019) | 1 | 1 | 1 | 1 | 1 | 1 | 1 | 0 | 1 | 8 |
