# Supplementary material for: Early Initiation of Breastfeeding and Exclusive Breastfeeding in Anglophone and Francophone West African Countries: Systematic Review and Meta‐Analysis of Prevalence
Source: Matern Child Nutr. 2025 Jan 7;21(2):e13792. doi: 10.1111/mcn.13792 (PMC11956053; doi:10.1111/mcn.13792)
Supplement: Supplementary file 5 — S4 Table. Prevalence of EIBF among Anglophone and Francophone West African countries. [file MCN-21-e13792-s006.docx]

**S4 Table. Prevalence of early initiation of breastfeeding in Anglophone and Francophone West African countries**

| **No** | **Author (Year)** | **Study setting** | **Subsector Category** | **Type of study** | **Population** | **Sample size** | **Prevalence (%)** |
| --- | --- | --- | --- | --- | --- | --- | --- |
| 1 | Olorunsaiye et al (2020) | Senegal | Francophone | Cross-sectional study | Women 15 to 49yrs with recent live birth within 3 yrs prior to the survey | 6328 | 37.7 |
| 2 | Sadoh et al (2011) | Nigeria | Anglophone | Cross-sectional study | 25-39 yr old female medical doctors below the level of consultants who had had a baby within the preceding 24 months and who had resumed work. | 36 | 13 |
| 3 | Appiah et al (2021) | Ghana | Anglophone | Community-based descriptive cross-sectional | Mothers with children <5yrs | 392 | 61.1 |
| 4 | Senbanjo et al (2014) | Nigeria | Anglophone | Cross-sectional study | 12-49 yr old Mothers with children 6-24months attending paediatric clinic | 311 | 27 |
| 5 | Atimati et al (2020) | Nigeria | Anglophone | Cross-sectional  descriptive study | Mother (<50yrs) child pairs with child 1-24 months old | 418 | 44.5 |
| 6 | Asare et al (2018) | Ghana | Anglophone | Cross-sectional descriptive study | 15-49yr Mothers with babies < 24months visiting a child welfare clinic | 355 | 63.4 |
| 7 | Olorunsaiye et al (2020) | Nigeria | Anglophone | Cross-sectional study | Women (15-49yr) with live birth within three years prior to the survey | 16077 | 34.3 |
| 8 | Gebremedhin (2019) | Gambia | Anglophone | Descriptive cross-sectional study | Women (15-49yr) in the reproductive age who gave at least one birth in the preceding 24 months of the survey. | 372 | 51.4 |
|  |  | Ghana | Anglophone |  |  | 5043 | 53.9 |
|  |  | Liberia | Anglophone |  |  | 744 | 60.6 |
|  |  | Nigeria | Anglophone |  |  | 33398 | 33.2 |
|  |  | Sierra Leone | Anglophone |  |  | 1198 | 52.7 |
|  |  | Benin | Francophone |  |  | 1983 | 49.2 |
|  |  | Burkina Faso | Francophone |  |  | 3394 | 41.8 |
|  |  | Cote d’Ivoire, | Francophone |  |  | 4254 | 29.7 |
|  |  | Guinea | Francophone |  |  | 2011 | 16.6 |
|  |  | Mali | Francophone |  |  | 3274 | 55.7 |
|  |  | Niger | Francophone |  |  | 3473 | 52.3 |
|  |  | Senegal | Francophone |  |  | 2603 | 29 |
|  |  | Togo | Francophone |  |  | 1300 | 59.2 |
| 9 | Sackey et al (2023) | Ghana | Anglophone | Cross-sectional study | Mothers with children < -24 months | 257 | 78.2 |
| 10 | Onwuka (2022) | Nigeria | Anglophone | Cross-sectional study | Breastfeeding mothers with single babies < 6 months | 315 | 87 |
| 11 | Wan et al (2023) | Gambia | Anglophone | Cross-sectional study | Mothers (15-49yr) who delivered at a healthcare facility who have had children within 5yrs of survey | 7581 | 37.6 |
|  |  | Liberia | Anglophone |  |  | 5235 | 66.6 |
|  |  | Nigeria | Anglophone |  |  | 33711 | 51.9 |
|  |  | Sierra Leone | Anglophone |  |  | 9779 | 75.9 |
|  |  | Benin | Francophone |  |  | 13446 | 54.5 |
|  |  | Guinea | Francophone |  |  | 7890 | 53.2 |
|  |  | Mali | Francophone |  |  | 10286 | 69.5 |
|  |  | Senegal | Francophone |  |  | 5600 | 37.7 |
| 12 | Berde et al (2016) | Nigeria | Anglophone | Cross-sectional study | Mothers (15-49yr) with last born child born in the past 2yrs preceding survey | 11851 | 34.7 |
| 13 | Darboe et al (2023) | Gambia | Anglophone | Cross-sectional study | Women between 15 to 49 yrs with children <2yrs old | 5691 | 64.3 |
| 14 | Armah-Ansah et al (2023) | Benin | Francophone | Cross-sectional study | Women aged 15 -49 yr with children <2yrs | 7223 | 56 |
| 15 | Ezeh et al (2019) | Gambia | Anglophone | Cross-sectional study | Mothers with last born child at 23 months and living with the respondent | 5387 | 53 |
|  |  | Ghana | Anglophone |  |  | 3412 | 57 |
|  |  | Liberia | Anglophone |  |  | 4241 | 62 |
|  |  | Nigeria | Anglophone |  |  | 15993 | 34 |
|  |  | Sierra Leone | Anglophone |  |  | 6230 | 55 |
|  |  | Benin | Francophone |  |  | 5937 | 55 |
|  |  | Burkina Faso | Francophone |  |  | 6887 | 42 |
|  |  | Cote d’Ivoire, | Francophone |  |  | 4554 | 32 |
|  |  | Guinea | Francophone |  |  | 3574 | 17 |
|  |  | Mali | Francophone |  |  | 4843 | 59 |
|  |  | Niger | Francophone |  |  | 7044 | 54 |
|  |  | Senegal | Francophone |  |  | 5790 | 34 |
|  |  | Togo | Francophone |  |  | 3042 | 62 |
| 16 | Nukpeza et al (2018) | Ghana | Anglophone | Descriptive Cross-sectional study | Mother-infant pairs attending child welfare clinics with children <2yrs | 393 | 39.4 |
| 17 | Birhan et al (2022) | Gambia | Anglophone | Cross-sectional study | Women aged 15 -49 yr with children <2yrs | 7471 | 53.57 |
|  |  | Ghana | Anglophone |  |  | 5698 | 55.62 |
|  |  | Liberia | Anglophone |  |  | 7091 | 59.68 |
|  |  | Nigeria | Anglophone |  |  | 4051 | 74.47 |
|  |  | Sierra Leone | Anglophone |  |  | 10964 | 51.92 |
|  |  | Benin | Francophone |  |  | 12159 | 55.15 |
|  |  | Burkina Faso | Francophone |  |  | 14662 | 43.26 |
|  |  | Cote d’Ivoire, | Francophone |  |  | 7258 | 23.58 |
|  |  | Guinea | Francophone |  |  | 7453 | 45.09 |
|  |  | Mali | Francophone |  |  | 8795 | 66.54 |
|  |  | Niger | Francophone |  |  | 11460 | 56.63 |
|  |  | Senegal | Francophone |  |  | 17426 | 27.85 |
|  |  | Togo | Francophone |  |  | 9129 | 53.34 |
| 18 | Appiah et al (2021) | Gambia | Anglophone | Cross-sectional study | Women aged 15–49 years with birth history and who had children born in the 2 years preceding the survey and practiced breastfeeding | 1596 | 55.91 |
|  |  | Ghana | Anglophone |  |  | 1171 | 57.47 |
|  |  | Liberia | Anglophone |  |  | 1281 | 63.56 |
|  |  | Sierra Leone | Anglophone |  |  | 2108 | 58.84 |
|  |  | Benin | Francophone |  |  | 4413 | 54.32 |
|  |  | Burkina Faso | Francophone |  |  | 2909 | 43.86 |
|  |  | Guinea | Francophone |  |  | 1429 | 15.17 |
|  |  | Mali | Francophone |  |  | 2138 | 58 |
|  |  | Niger | Francophone |  |  | 2334 | 51.62 |
|  |  | Senegal | Francophone |  |  | 1710 | 50.04 |
|  |  | Togo | Francophone |  |  | 838 | 58.47 |
| 19 | Gyan Aboagye et al (2023) | Gambia | Anglophone | Cross-sectional study | Women15 -49yrs who had given birth 2 years preceding the survey | 1595 | 36.33 |
|  |  | Liberia | Anglophone |  |  | 1030 | 67.64 |
|  |  | Nigeria | Anglophone |  |  | 4899 | 45.12 |
|  |  | Sierra Leone | Anglophone |  |  | 1760 | 79.77 |
|  |  | Benin | Francophone |  |  | 5212 | 56.04 |
|  |  | Guinea | Francophone |  |  | 1375 | 39.86 |
|  |  | Mali | Francophone |  |  | 3901 | 65.9 |
| 20 | Dubik et al (2021) | Ghana | Anglophone | Cross-sectional study | Mothers with infants aged 0–24 months in the Sagnarigu Municipality of Northern Ghana. | 508 | 72 |
| 21 | Apanga et al (2021) | Ghana | Anglophone | Cross-sectional study | Women of reproductive age (15–49 years) with a live birth within 2 years. | 3466 | 52.3 |
| 22 | Issaka et al (2017) | Gambia | Anglophone | Cross-sectional study | Mothers with children 6-23 months | 4886 | 52.55 |
|  |  | Ghana | Anglophone |  |  | 5710 | 56.58 |
|  |  | Liberia | Anglophone |  |  | 2839 | 62.34 |
|  |  | Nigeria | Anglophone |  |  | 3275 | 33.86 |
|  |  | Sierra Leone | Anglophone |  |  | 2202 | 55.14 |
|  |  | Benin | Francophone |  |  | 2672 | 51.25 |
|  |  | Burkina Faso | Francophone |  |  | 2467 | 42.36 |
|  |  | Cote d’Ivoire, | Francophone |  |  | 3802 | 31.74 |
|  |  | Guinea | Francophone |  |  | 11712 | 16.54 |
|  |  | Mali | Francophone |  |  | 4939 | 58.68 |
|  |  | Niger | Francophone |  |  | 4316 | 53.65 |
|  |  | Senegal | Francophone |  |  | 4327 | 48.61 |
| 23 | Anaba et al (2022) | Nigeria | Anglophone | Cross-sectional study | 15-49yr women with a child under 2 years of age | 3039 | 42.1 |
| 24 | Adewuyi et al (2017) | Nigeria | Anglophone | Cross-sectional study | Mothers who provided information on their last life child <2yrs | 11851 | 36.4 |
| 25 | Ekholuenetale et al (2022) | Nigeria | Anglophone | Cross-sectional study |  |  | 44.2 |
| 26 | Teshale et al (2021) | Benin | Francophone | Cross-sectional study | Women (15-49yr) with child <2yrs | 10309 | 55.93 |
|  |  | Burkina Faso | Francophone |  |  | 5739 | 43.93 |
|  |  | Cote d’Ivoire, | Francophone |  |  | 2923 | 26.69 |
|  |  | Gambia | Anglophone |  |  | 1585 | 36.13 |
|  |  | Ghana | Anglophone |  |  | 1153 | 57.36 |
|  |  | Guinea | Francophone |  |  | 1358 | 40.24 |
|  |  | Liberia | Anglophone |  |  | 1266 | 63.28 |
|  |  | Mali | Francophone |  |  | 3850 | 66.06 |
|  |  | Nigeria | Anglophone |  |  | 4801 | 45.3 |
|  |  | Niger | Francophone |  |  | 2351 | 51.78 |
|  |  | Senegal | Francophone |  |  | 2340 | 29.84 |
|  |  | Sierra Leone | Anglophone |  |  | 2117 | 58.71 |
|  |  | Togo | Francophone |  |  | 1312 | 62.47 |
| 27 | Boakye-Yiadom et al (2021) | Ghana | Anglophone | Cross-sectional study | Newly delivered mothers at KATH who had live births and whose infants were alive after 24 h. | 376 | 39.4 |
| 28 | Ekholuenetale et al (2021) | Benin | Francophone | Cross-sectional study | Women (15-49yr) with child <2yrs | 13407 | 50.4 |
|  |  | Burkina Faso | Francophone |  |  | 15044 | 42.1 |
|  |  | Cote d’Ivoire, | Francophone |  |  | 7776 | 30.8 |
|  |  | Gambia | Anglophone |  |  | 8088 | 51.5 |
|  |  | Ghana | Anglophone |  |  | 5884 | 55.6 |
|  |  | Guinea | Francophone |  |  | 7039 | 16.6 |
|  |  | Liberia | Anglophone |  |  | 7606 | 61.2 |
|  |  | Mali | Francophone |  |  | 10326 | 57.8 |
|  |  | Nigeria | Anglophone |  |  | 31482 | 33.2 |
|  |  | Niger | Francophone |  |  | 12558 | 52.9 |
|  |  | Senegal | Francophone |  |  | 12185 | 33.6 |
|  |  | Sierra Leone | Anglophone |  |  | 11938 | 53.8 |
|  |  | Togo | Francophone |  |  | 6979 | 60.6 |
| 29 | Ogbo et al (2015) | Nigeria | Anglophone | Cross-sectional study | 15 -49 yrs Women living with youngest living child < 24 months | 10225 | 37.7 |
| 30 | Ogbo et al (2017) | Burkina Faso | Francophone | Cross-sectional study | Mother with children under 24 months | 5710 | 42 |
|  |  | Mali | Francophone |  |  | 3802 | 59 |
|  |  | Nigeria | Anglophone |  |  | 11712 | 34 |
|  |  | Niger | Francophone |  |  | 4930 | 54 |
| 31 | Ogunlesi, T. A (2010) | Nigeria | Anglophone | Cross-sectional study | Mothers (18-43yrs) of children 1-24 months attending infant welfare clinic | 262 | 37.4 |
| 32 | Okafor et al (2014) | Nigeria | Anglophone | Cross-sectional study | Mother (15 - 45yr) with children under 24 months | 600 | 59.2 |
| 33 | Akadri et al (2020) | Nigeria | Anglophone | Cross-sectional study | Non primiparous Pregnant Women from antenatal clinics of 2 teaching hospitals who practiced breastfeeding in previous pregnancy | 340 | 38.8 |
| 34 | Morhason-Bello et al (2022) | Nigeria | Anglophone | Cross-sectional study | 15-49yr old women who have had a childbirth in the past 5 years preceding the surveys. | 11450 | 31.5 |
|  |  | Nigeria | Anglophone |  |  | 12349 | 37.6 |
|  |  | Nigeria | Anglophone |  |  | 16990 | 35.3 |
|  |  | Nigeria | Anglophone |  |  | 17455 | 43.8 |
| 35 | Setorglo et al (2020) | Ghana | Anglophone | Descriptive cross-sectional study | Nursing mothers with babies 6-24 months attending clinics | 391 | 80 |
| 36 | Cresswell et al (2017) | Burkina Faso | Francophone | Cross-sectional study | Women (15 to 49 years) with at least one live birth< 12 months | 2288 | 9.4 |
| 37 | Abdul-Aziz et al (2020) | Ghana | Anglophone | Cross-sectional study | Mothers 15-49yrs with children <2yrs preceding the survey | 4219 | 55.7 |
| 38 | Duodu et al (2021) | Ghana | Anglophone | Cross-sectional study | Mothers and infant pairs 15-49yrs mothers and 0-5yrs children | 15319 | 55.1 |
| 39 | Haile et al (2018) | Ghana | Anglophone | Cross-sectional study | Women 15-49yrs who had a live birth in the 5 years preceding the 2014 survey | 3087 | 58.3 |
| 40 | Kim et al, (2023) | Burkina Faso | Francophone | Cross-sectional study | Women (average 27yrs) recently delivered with child <6mths | 1840 | 40 |
