# Supplementary material for: Early Initiation of Breastfeeding and Exclusive Breastfeeding in Anglophone and Francophone West African Countries: Systematic Review and Meta‐Analysis of Prevalence
Source: Matern Child Nutr. 2025 Jan 7;21(2):e13792. doi: 10.1111/mcn.13792 (PMC11956053; doi:10.1111/mcn.13792)
Supplement: Supplementary file 6 — S5 Table. Prevalence of EBF among Anglophone and Francophone West African countries. [file MCN-21-e13792-s003.docx]

**S5 Table. Prevalence of exclusive breastfeeding in Anglophone and Francophone West Africa**

|  | **Author (Year)** | **Study setting** | **Subsector Category** | **Type of study** | **Population** | **Sample size** | **Prevalence EBF (%)** |
| --- | --- | --- | --- | --- | --- | --- | --- |
| 1 | Sadoh et al (2011) | Nigeria | Anglophone | Cross-sectional study | 25-39 yr old female medical doctors below the level of consultants who had had a baby within the preceding 24 months and who had resumed work. | 36 | 11.1 |
| 2 | Appiah et al (2021) | Ghana | Anglophone | Community-based descriptive cross-sectional | Mothers with children <5yrs | 396 | 43.7 |
| 3 | Senbanjo et al (2014) | Nigeria | Anglophone | Cross-sectional study | 12-49 yr old Mothers with children 6-24months attending paediatric clinic | 311 | 36 |
| 4 | Atimati et al (2020) | Nigeria | Anglophone | Cross-sectional descriptive study | Mother (<50yrs) child pairs with child 1-24 months old | 418 | 36.6 |
| 5 | Asare et al (2018) | Ghana | Anglophone | Cross-sectional descriptive study | 15-49yr Mothers with babies < 24months visiting a child welfare clinic | 355 | 66 |
| 6 | Diji et al (2016) | Ghana | Anglophone | Descriptive Cross-sectional study | Mothers (19-39yrs) with babies <9 months attending child welfare clinic | 240 | 66.7 |
| 7 | Gebremedhin (2019) | Gambia | Anglophone | Descriptive cross-sectional study | Women (15-49yr) in the reproductive age who gave at least one birth in the preceding 24 months of the survey. | 372 | 46.5 |
|  |  | Ghana | Anglophone |  |  | 5043 | 52.3 |
|  |  | Liberia | Anglophone |  |  | 744 | 56 |
|  |  | Nigeria | Anglophone |  |  | 33398 | 17 |
|  |  | Sierra Leone | Anglophone |  |  | 1198 | 32.3 |
|  |  | Benin | Francophone |  |  | 1983 | 32.1 |
|  |  | Burkina Faso | Francophone |  |  | 3394 | 24.7 |
|  |  | Cote d’Ivoire | Francophone |  |  | 4254 | 12.1 |
|  |  | Guinea | Francophone |  |  | 2011 | 20.5 |
|  |  | Mali | Francophone |  |  | 3274 | 32.9 |
|  |  | Niger | Francophone |  |  | 3473 | 23.2 |
|  |  | Senegal | Francophone |  |  | 2603 | 26.2 |
|  |  | Togo | Francophone |  |  | 1300 | 57.6 |
| 8 | Hitachi et al (2019) | Niger | Francophone | Community based cross-sectional study | Urban and 283 rural mothers of infants <7mths old | 517 | 34.6 |
| 9 | Onwuka (2022) | Nigeria | Anglophone | Cross-sectional study | Breastfeeding mothers with single babies < 6 months | 315 | 69 |
| 10 | Yalçin et al (2016) | Ghana | Anglophone | Cross-sectional study | Women (15-49yr) with infants <6months | 317 | 63.1 |
|  |  | Liberia | Anglophone |  |  | 497 | 29.8 |
|  |  | Nigeria | Anglophone |  |  | 2833 | 37 |
|  |  | Sierra Leone | Anglophone |  |  | 590 | 11.7 |
|  |  | Benin | Francophone |  |  | 1118 | 35.2 |
|  |  | Burkina Faso | Francophone |  |  | 1452 | 24.8 |
|  |  | Cote d’Ivoire, | Francophone |  |  | 770 | 12.3 |
|  |  | Guinea | Francophone |  |  | 714 | 20.7 |
|  |  | Mali | Francophone |  |  | 1418 | 37.8 |
|  |  | Niger | Francophone |  |  | 1299 | 23.3 |
|  |  | Senegal | Francophone |  |  | 1318 | 39.3 |
| 11 | Tampah-Naah et al (2013) | Ghana | Anglophone | Cross-sectional study | Mothers of Children 0-5months | 316 | 64 |
| 12 | Agho et al (2011) | Nigeria | Anglophone | Cross-sectional study | Mothers of Children < 6 months | 658 | 16.4 |
| 13 | Olasinde et al (2021) | Nigeria | Anglophone | Cross-sectional  Descriptive study | Mothers of infants aged less than six months attending immunization clinic | 271 | 46.1 |
| 14 | Soumah et al (2021) | Guinea | Francophone | Cross-sectional study | Women aged 15 to 49 with their last birth 6 months prior to the data collection | 851 | 33 |
| 15 | Ogbo et al (2015) | Nigeria | Anglophone | Cross-sectional study | 15 -49 yrs Women living with youngest living child < 24 months | 10225 | 13.5 |
| 16 | Manyeh et al (2020) | Ghana | Anglophone | Cross-sectional study | Mothers with the index baby at least 6months registered with DHDSS | 1870 | 71 |
| 17 | Dun-Dery et al (2016) | Ghana | Anglophone | Descriptive  cross-sectional study | City dwelling professional women >24yrs with child 6-24 months old | 369 | 10.3 |
| 18 | Gayawan et al (2014) | Nigeria | Anglophone | Cross-sectional study | Mothers with Infants between 0-5 months | 4113 | 12.6 |
| 19 | Yeboah et al (2019) | Ghana | Anglophone | Cross-sectional study | Lactating mothers with child 6 - 24 months | 160 | 50.6 |
| 20 | Agho et al (2019) | Gambia | Anglophone | Cross-sectional study | Mothers with last born child aged 0–5 months and living with the respondent | 1422 | 47 |
|  |  | Ghana | Anglophone |  |  | 805 | 52 |
|  |  | Liberia | Anglophone |  |  | 927 | 55 |
|  |  | Nigeria | Anglophone |  |  | 3996 | 17 |
|  |  | Sierra Leone | Anglophone |  |  | 1842 | 32 |
|  |  | Benin | Francophone |  |  | 1475 | 44 |
|  |  | Burkina Faso | Francophone |  |  | 1837 | 25 |
|  |  | Cote d’Ivoire, | Francophone |  |  | 1110 | 13 |
|  |  | Guinea | Francophone |  |  | 981 | 21 |
|  |  | Mali | Francophone |  |  | 1192 | 34 |
|  |  | Niger | Francophone |  |  | 2196 | 24 |
|  |  | Senegal | Francophone |  |  | 1298 | 44 |
|  |  | Togo | Francophone |  |  | 655 | 58 |
| 21 | Terefe et al (2023) | Gambia | Anglophone | Cross-sectional study | Mothers with children 0-6months | 897 | 53.63 |
| 22 | Dadzie et al (2023) | Ghana | Anglophone | Quantitative Cross-sectional study | 16 – 45yr Mothers with children between 6 – 24 months visiting a child welfare clinic | 222 | 68.8 |
| 23 | Nukpeza et al (2018) | Ghana | Anglophone | Descriptive Cross-sectional study | Mother-infant pairs attending child welfare clinics with children <2yrs | 393 | 27.7 |
| 24 | Oche et al (2011) | Nigeria | Anglophone | Descriptive Cross-sectional study | Breastfeeding women /stopped breastfeeding within past 2 yrs | 179 | 31 |
| 25 | Osibogun et al (2018) | Nigeria | Anglophone | Cross-sectional descriptive study | Mothers with at least one child | 200 | 28.5 |
| 26 | Sokan-Adeaga et al 2022 | Nigeria | Anglophone | Cross-sectional study | Mothers aged 15 to 49 years who are currently breastfeeding infants between 0 to 24 months and who are attending postnatal clinic | 120 | 68.3 |
| 27 | Adebayo et al (2021) | Nigeria | Anglophone | Cross-sectional study | Nursing mothers attending the immunization clinic at medical centre whose baby <2yrs | 386 | 52.6 |
| 28 | Duarte et al (2022) | Cape Verde | Francophone | Quantitative, descriptive and cross-sectional | Women with children <2yrs who attended health facility for postnatal care | 1717 | 32.5 |
| 29 | Issaka et al (2017) | Benin | Francophone | Cross-sectional study | Mothers with children 6-23 months | 1154 | 33.97 |
|  |  | Burkina Faso | Francophone |  |  | 1504 | 24.87 |
|  |  | Cote d’Ivoire, | Francophone |  |  | 730 | 13.15 |
|  |  | Gambia | Anglophone |  |  | 913 | 46.77 |
|  |  | Ghana | Anglophone |  |  | 561 | 52.58 |
|  |  | Guinea | Francophone |  |  | 728 | 20.6 |
|  |  | Liberia | Anglophone |  |  | 590 | 55.25 |
|  |  | Mali | Francophone |  |  | 974 | 33.68 |
|  |  | Nigeria | Anglophone |  |  | 2926 | 17.5 |
|  |  | Niger | Francophone |  |  | 1480 | 23.38 |
|  |  | Senegal | Francophone |  |  | 1179 | 38.85 |
|  |  | Sierra Leone | Anglophone |  |  | 1208 | 32.12 |
| 30 | Anaba et al (2022) | Nigeria | Anglophone | Cross-sectional study | 15-49yr women with a child under 2 years of age | 721 | 37.5 |
| 31 | Ekholuenetale et al (2022) | Nigeria | Anglophone | Cross-sectional study | Children on DHS data | 2936 | 31.8 |
| 32 | Mohammed et al (2022) | Ghana | Anglophone | Cross-sectional study | Mothers with children 0-5months | 3329 | 51.6 |
| 33 | Ogbo et al (2017) | Burkina Faso | Francophone | Cross-sectional study | Mother with children under 24 months | 5710 | 25 |
|  |  | Mali | Francophone |  |  | 3802 | 34 |
|  |  | Nigeria | Anglophone |  |  | 11712 | 17 |
|  |  | Niger | Francophone |  |  | 4930 | 23 |
| 34 | Ogunlesi, T. A (2010) | Nigeria | Anglophone | Cross-sectional study | Mothers (18-43yrs) of children 1-24 months attending infant welfare clinic | 262 | 21.4 |
| 35 | Okafor et al (2014) | Nigeria | Anglophone | Cross-sectional study | Mother (15 - 45yr) with children under 24 months | 600 | 52.9 |
| 36 | Adamu et al (2022) | Nigeria | Anglophone | Descriptive Cross-sectional study | Mothers of children 6 -24 months attending paediatric follow up and family health clinic | 240 | 34.2 |
| 37 | Akadri et al (2020) | Nigeria | Anglophone | Cross-sectional study | Non primiparous Pregnant Women from antenatal clinics of 2 teaching hospitals who practiced breastfeeding in previous pregnancy | 340 | 58.8 |
| 38 | Anyanwu et al (2014) | Nigeria | Anglophone | Cross-sectional descriptive study | Female healthcare workers who have children and who have worked at least 2 yrs in a tertiary hospital | 100 | 25 |
| 39 | Yakubu et al (2023) | Nigeria | Anglophone | Descriptive cross-sectional study | Nursing mothers (20 -50yrs) with babies less than 2 yrs old | 230 | 70 |
| 40 | Uchendu et al (2009) | Nigeria | Anglophone | Cross-sectional study | Women with children > 6 months attending a paedics clinic | 184 | 32.4 |
| 41 | Setorglo et al (2020) | Ghana | Anglophone | Cross-sectional study | Nursing mothers with babies 6-24 months attending clinics | 391 | 58.8 |
| 42 | Cresswell et al (2017) | Burkina Faso | Francophone | Cross-sectional study | Women (15 to 49 years) with at least one live birth< 12 months | 2288 | 30 |
| 43 | Koffi et al (2023) | Cote d’Ivoire, | Francophone | Cross-sectional study | Mothers with children aged 0-5mths | 980 | 23.5 |
| 44 | Kim et al, (2023) | Burkina Faso | Francophone | Cross-sectional study | Women (average 27yrs) recently delivered with child <6mths | 1840 | 65 |
