# Supplementary material for: Early Initiation of Breastfeeding and Exclusive Breastfeeding in Anglophone and Francophone West African Countries: Systematic Review and Meta‐Analysis of Prevalence
Source: Matern Child Nutr. 2025 Jan 7;21(2):e13792. doi: 10.1111/mcn.13792 (PMC11956053; doi:10.1111/mcn.13792)
Supplement: Supplementary file 7 — S6 Table. Selection strategy for one database. [file MCN-21-e13792-s008.docx]

**S3: Full Search strategy (Prevalence) for one database**

Cinahl with full text (via Ebsco host)

AB (Breastfeed* or "Breast feed*" or "Infant feed*" or Feed* or "Early Initiat*" or "Exclusive breast feed*" or "Breastfeeding Indic*” )

AND

TX (Prevalence* or rate* )

AND

TX (Gambia or Ghana or Liberia or Nigeria or "Sierra Leone" or Benin or "Burkina Faso" or "Cape Verde" or "Cote d’Ivoire" or Guinea or "Guinea Bissau" or Mali or Niger or Senegal or Togo or "Sub Sahara*" or ECOWAS or "West Africa*” )

Limits

Full Text; Publication Date: 20080101-20240331; English Language; Peer Reviewed
