# Supplementary material for: Early Initiation of Breastfeeding and Exclusive Breastfeeding in Anglophone and Francophone West African Countries: Systematic Review and Meta‐Analysis of Prevalence
Source: Matern Child Nutr. 2025 Jan 7;21(2):e13792. doi: 10.1111/mcn.13792 (PMC11956053; doi:10.1111/mcn.13792)
Supplement: Supplementary file 8 — S7 Table. Characteristics of included studies. [file MCN-21-e13792-s004.docx]

**Table 1: Characteristics of studies included in the review of EIBF and EBF**

| **No** | **Author (Year)** | **Study setting** | **Subsector** | **Sample size (N)** | | **Population Characteristics** | **Sampling strategy** | **Study design** | **Quality assessment** |
| --- | --- | --- | --- | --- | --- | --- | --- | --- | --- |
|  |  |  |  | **EIBF** | **EBF** |  |  |  |  |
| 1 | Abdul-Aziz et al (2020) | Ghana | Anglophone | 4219 |  | Mothers 15-49yrs with children <2yrs preceding the survey | Two-stage sampling | Cross-sectional study | High |
| 2 | Adamu et al (2022) | Nigeria | Anglophone |  | 240 | Mothers of children 6 -24 months attending paediatric follow up and family health clinic | Consecutive sampling | Descriptive cross‑sectional study | High |
| 3 | Adebayo et al (2021) | Nigeria | Anglophone |  | 386 | Nursing mothers attending the immunization clinic at medical centre whose baby <2yrs | Systematic random sampling | Cross-sectional study | High |
| 4 | Adewuyi et al (2017) | Nigeria | Anglophone | 11851 |  | Mothers who provided information on their last life child <2yrs | Stratified three-stage cluster sampling | Cross-sectional study | High |
| 5 | Agho et al (2011) | Nigeria | Anglophone |  | 658 | Mothers of Children < 6 months | Multi-stage cluster sample | Cross-sectional study | High |
| 6 | Agho et al (2019) | Gambia | Anglophone |  | 1422 | Mothers with last born child aged 0–5 months and living with the respondent | Multistage cluster sample | Cross-sectional study | High |
|  |  | Ghana | Anglophone |  | 805 |  |  |  |  |
|  |  | Liberia | Anglophone |  | 927 |  |  |  |  |
|  |  | Nigeria | Anglophone |  | 3996 |  |  |  |  |
|  |  | Sierra Leone | Anglophone |  | 1842 |  |  |  |  |
|  |  | Benin | Francophone |  | 1475 |  |  |  |  |
|  |  | Burkina Faso | Francophone |  | 1837 |  |  |  |  |
|  |  | Cote d’Ivoire | Francophone |  | 1110 |  |  |  |  |
|  |  | Guinea | Francophone |  | 981 |  |  |  |  |
|  |  | Mali | Francophone |  | 1192 |  |  |  |  |
|  |  | Niger | Francophone |  | 2196 |  |  |  |  |
|  |  | Senegal | Francophone |  | 1298 |  |  |  |  |
|  |  | Togo | Francophone |  | 655 |  |  |  |  |
| 7 | Akadri et al (2020) | Nigeria | Anglophone | 340 | 340 | Non primiparous Pregnant Women from antenatal clinics of 2 teaching hospitals who practiced breastfeeding in previous pregnancy | Consecutive sampling | Cross-sectional study | High |
| 8 | Anaba et al (2022) | Nigeria | Anglophone | 3039 | 721 | 15-49yr women with a child under 2 years of age | Two‐stage cluster‐sample | Cross-sectional study | High |
| 9 | Anyanwu et al (2014) | Nigeria | Anglophone |  | 100 | Female healthcare workers who have children and who have worked at least 2 yrs in a tertiary hospital | Convenience sampling | Cross-sectional descriptive | Medium |
| 10 | Apanga et al (2021) | Ghana | Anglophone | 3466 |  | Women of reproductive age (15–49 years) with a live birth within 2 years. | Two-stage sampling | Cross-sectional study | High |
| 11 | Appiah et al (2021) | Ghana | Anglophone | 396 | 396 | Mothers with children <5yrs | Multi-stage cluster sampling | Community-based descriptive cross-sectional | High |
| 12 | Appiah et al (2021) | Gambia | Anglophone | 1596 |  | Women aged 15–49 years with birth history and who had children born in the 2 years preceding the survey and practiced breastfeeding | Two-stage stratified sampling | Cross-sectional study | High |
|  |  | Ghana | Anglophone | 1171 |  |  |  |  |  |
|  |  | Liberia | Anglophone | 1281 |  |  |  |  |  |
|  |  | Sierra Leone | Anglophone | 2108 |  |  |  |  |  |
|  |  | Benin | Francophone | 4413 |  |  |  |  |  |
|  |  | Burkina Faso | Francophone | 2909 |  |  |  |  |  |
|  |  | Guinea | Francophone | 1429 |  |  |  |  |  |
|  |  | Mali | Francophone | 2138 |  |  |  |  |  |
|  |  | Niger | Francophone | 2334 |  |  |  |  |  |
|  |  | Senegal | Francophone | 1710 |  |  |  |  |  |
|  |  | Togo | Francophone | 838 |  |  |  |  |  |
| 13 | Armah-Ansah et al (2023) | Benin | Francophone | 7223 |  | Women aged 15 -49 yr with children <2yrs | Stratified stage cluster design | Cross-sectional study | High |
| 14 | Asare et al (2018) | Ghana | Anglophone | 355 | 355 | 15-49yr Mothers with babies < 24months visiting a child welfare clinic | Non random sampling | Cross-sectional descriptive | High |
| 15 | Atimati et al (2020) | Nigeria | Anglophone | 418 | 418 | Mother (<50yrs) child pairs with child 1-24 months old | Multistage sampling | Cross-sectional descriptive | High |
| 16 | Berde et al (2016) | Nigeria | Anglophone | 11851 |  | Mothers (15-49yr) with last born child born in the past 2yrs preceding survey | Stratified three-stage cluster design | Cross-sectional study | High |
| 17 | Bergamaschi et al (2019) | Benin | Francophone | Not reported |  | Mothers (15-49yr) with last born child born in the past 2yrs preceding survey | Stratified multi-stage cluster sampling | Cross-sectional study | High |
|  |  | Burkina Faso | Francophone |  |  |  |  |  |  |
|  |  | Ghana | Anglophone |  |  |  |  |  |  |
|  |  | Guinea | Francophone |  |  |  |  |  |  |
|  |  | Liberia | Anglophone |  |  |  |  |  |  |
|  |  | Mali | Francophone |  |  |  |  |  |  |
|  |  | Niger | Francophone |  |  |  |  |  |  |
|  |  | Nigeria | Anglophone |  |  |  |  |  |  |
|  |  | Senegal | Francophone |  |  |  |  |  |  |
|  |  | Sierra Leone | Anglophone |  |  |  |  |  |  |
| 18 | Birhan et al (2022) | Gambia | Anglophone | 7471 |  | Women aged 15 -49 yr with children <2yrs | Stratified, multi-stage, random sampling | Cross-sectional study | High |
|  |  | Ghana | Anglophone | 5698 |  |  |  |  |  |
|  |  | Liberia | Anglophone | 7091 |  |  |  |  |  |
|  |  | Nigeria | Anglophone | 4051 |  |  |  |  |  |
|  |  | Sierra Leone | Anglophone | 10964 |  |  |  |  |  |
|  |  | Benin | Francophone | 12159 |  |  |  |  |  |
|  |  | Burkina Faso | Francophone | 14662 |  |  |  |  |  |
|  |  | Cote d’Ivoire, | Francophone | 7258 |  |  |  |  |  |
|  |  | Guinea | Francophone | 7453 |  |  |  |  |  |
|  |  | Mali | Francophone | 8795 |  |  |  |  |  |
|  |  | Niger | Francophone | 11460 |  |  |  |  |  |
|  |  | Senegal | Francophone | 17426 |  |  |  |  |  |
|  |  | Togo | Francophone | 9129 |  |  |  |  |  |
| 19 | Boakye-Yiadom et al (2021) | Ghana | Anglophone | 376 |  | Newly delivered mothers at KATH who had live births and whose infants were alive after 24 h. | Systematic random sampling | Cross-sectional study | High |
| 20 | Cresswell et al (2017) | Burkina Faso | Francophone | 2288 | 2288 | Women (15 to 49 years) with at least one live birth< 12 months | Stratified two-stage design | Cross-sectional study | High |
| 21 | Dadzie et al (2023) | Ghana | Anglophone |  | 222 | 16 – 45yr Mothers with children between 6 – 24 months visiting a child welfare clinic | Random sampling | Quantitative cross-sectional | High |
| 22 | Darboe et al (2023) | Gambia | Anglophone | 5691 |  | Women between 15 to 49 yrs with children <2yrs old | Two-stage sampling | Cross-sectional study | High |
| 23 | Diji et al (2016) | Ghana | Anglophone |  | 240 | Mothers (19-39yrs) with babies <9 months attending child welfare clinic | Simple random sampling | Descriptive cross-sectional | High |
| 24 | Duarte et al (2022) | Cape Verde | Francophone |  | 1717 | Women with children <2yrs who attended health facility for postnatal care | Probabilistic sample | Quantitative, descriptive and cross-sectional | High |
| 25 | Dubik et al (2021) | Ghana | Anglophone | 508 |  | Mothers with infants aged 0–24 months in the Sagnarigu Municipality of Northern Ghana. | Multi-stage sampling | Cross-sectional study | High |
| 26 | Dun-Dery et al (2016) | Ghana | Anglophone |  | 369 | City dwelling professional women >24yrs with child 6-24 mths old | Multi-stage random sampling | Descriptive cross-sectional | High |
| 27 | Duodu et al (2021) | Ghana | Anglophone | 15319 |  | Mothers and infant pairs 15-49yrs mothers and 0-5yrs children | Two-stage sampling | Cross-sectional study | High |
| 28 | Ekholuenetale et al (2022) | Nigeria | Anglophone | 21569 | 2936 | Children on DHS data | Stratified multistage cluster sampling | Cross-sectional study | High |
| 29 | Ekholuenetale et al (2021) | Benin | Francophone | 13407 |  | Women (15-49yr) with child <2yrs | Stratified multi-stage cluster sampling | Cross-sectional study | High |
|  |  | Burkina Faso | Francophone | 15044 |  |  |  |  |  |
|  |  | Cote d’Ivoire, | Francophone | 7776 |  |  |  |  |  |
|  |  | Gambia | Anglophone | 8088 |  |  |  |  |  |
|  |  | Ghana | Anglophone | 5884 |  |  |  |  |  |
|  |  | Guinea | Francophone | 7039 |  |  |  |  |  |
|  |  | Liberia | Anglophone | 7606 |  |  |  |  |  |
|  |  | Mali | Francophone | 10326 |  |  |  |  |  |
|  |  | Nigeria | Anglophone | 31482 |  |  |  |  |  |
|  |  | Niger | Francophone | 12558 |  |  |  |  |  |
|  |  | Senegal | Francophone | 12185 |  |  |  |  |  |
|  |  | Sierra Leone | Anglophone | 11938 |  |  |  |  |  |
|  |  | Togo | Francophone | 6979 |  |  |  |  |  |
| 30 | Ekholuenetale et al (2022) | Gambia | Anglophone |  | Not reported | women (15-49yr) with babies <6 months | multi-stage cluster stratified sampling | Cross sectional study | High |
|  |  | Ghana | Anglophone |  |  |  |  |  |  |
|  |  | Liberia | Anglophone |  |  |  |  |  |  |
|  |  | Nigeria | Anglophone |  |  |  |  |  |  |
|  |  | Sierra Leone | Anglophone |  |  |  |  |  |  |
|  |  | Benin | Francophone |  |  |  |  |  |  |
|  |  | Burkina Faso | Francophone |  |  |  |  |  |  |
|  |  | Cote d’Ivoire, | Francophone |  |  |  |  |  |  |
|  |  | Guinea | Francophone |  |  |  |  |  |  |
|  |  | Mali | Francophone |  |  |  |  |  |  |
|  |  | Niger | Francophone |  |  |  |  |  |  |
|  |  | Senegal | Francophone |  |  |  |  |  |  |
|  |  | Togo | Francophone |  |  |  |  |  |  |
| 31 | Ezeh et al (2019) | Gambia | Anglophone | 5387 |  | Mothers with last born child at 23 months and living with the respondent. | Stratified multistage cluster sampling | Cross-sectional study | High |
|  |  | Ghana | Anglophone | 3412 |  |  |  |  |  |
|  |  | Liberia | Anglophone | 4241 |  |  |  |  |  |
|  |  | Nigeria | Anglophone | 15993 |  |  |  |  |  |
|  |  | Sierra Leone | Anglophone | 6230 |  |  |  |  |  |
|  |  | Benin | Francophone | 5937 |  |  |  |  |  |
|  |  | Burkina Faso | Francophone | 6887 |  |  |  |  |  |
|  |  | Cote d’Ivoire, | Francophone | 4554 |  |  |  |  |  |
|  |  | Guinea | Francophone | 3574 |  |  |  |  |  |
|  |  | Mali | Francophone | 4843 |  |  |  |  |  |
|  |  | Niger | Francophone | 7044 |  |  |  |  |  |
|  |  | Senegal | Francophone | 5790 |  |  |  |  |  |
|  |  | Togo | Francophone | 3042 |  |  |  |  |  |
| 32 | Gayawan et al (2014) | Nigeria | Anglophone |  | 4113 | Mothers with Infants between 0-5 months | Two-stage stratified sampling | Cross-sectional study | High |
| 33 | Gebremedhin (2019) | Gambia | Anglophone | 372 | 372 | Women (15-49yr) in the reproductive age who gave at least one birth in the preceding 24 months of the survey. | Stratified two-stage design | Descriptive cross-sectional study | High |
|  |  | Ghana | Anglophone | 5043 | 5043 |  |  |  |  |
|  |  | Liberia | Anglophone | 744 | 744 |  |  |  |  |
|  |  | Nigeria | Anglophone | 33398 | 33398 |  |  |  |  |
|  |  | Sierra Leone | Anglophone | 1198 | 1198 |  |  |  |  |
|  |  | Benin | Francophone | 1983 | 1983 |  |  |  |  |
|  |  | Burkina Faso | Francophone | 3394 | 3394 |  |  |  |  |
|  |  | Cote d’Ivoire, | Francophone | 4254 | 4254 |  |  |  |  |
|  |  | Guinea | Francophone | 2011 | 2011 |  |  |  |  |
|  |  | Mali | Francophone | 3274 | 3274 |  |  |  |  |
|  |  | Niger | Francophone | 3473 | 3473 |  |  |  |  |
|  |  | Senegal | Francophone | 2603 | 2603 |  |  |  |  |
|  |  | Togo | Francophone | 1300 | 1300 |  |  |  |  |
| 34 | Gyan Aboagye et al (2023) | Gambia | Anglophone | 1595 |  | Women15 -49yrs who had given birth 2 years preceding the survey | Two-stage cluster sampling | Cross-sectional study | High |
|  |  | Liberia | Anglophone | 1030 |  |  |  |  |  |
|  |  | Nigeria | Anglophone | 4899 |  |  |  |  |  |
|  |  | Sierra Leone | Anglophone | 1760 |  |  |  |  |  |
|  |  | Benin | Francophone | 5212 |  |  |  |  |  |
|  |  | Guinea | Francophone | 1375 |  |  |  |  |  |
|  |  | Mali | Francophone | 3901 |  |  |  |  |  |
| 35 | Haile et al (2018) | Ghana | Anglophone | 3087 |  | Women 15-49yrs who had a live birth in the 5 years preceding the 2014 survey | Two-stage sampling | Cross-sectional study | High |
| 36 | Hitachi et al (2019) | Niger | Francophone |  | 517 | Urban and 283 rural mothers of infants <7mths old | Two stage sampling | Community-based cross-sectional study | High |
| 37 | Issaka et al (2017) | Benin | Francophone | 4886 | 1154 | Mothers with children 6-23 months | Two-stage cluster sampling | Cross-sectional study | High |
|  |  | Burkina Faso | Francophone | 5710 | 1504 |  |  |  |  |
|  |  | Cote d’Ivoire, | Francophone | 2839 | 730 |  |  |  |  |
|  |  | Gambia | Anglophone | 3275 | 913 |  |  |  |  |
|  |  | Ghana | Anglophone | 2202 | 561 |  |  |  |  |
|  |  | Guinea | Francophone | 2672 | 728 |  |  |  |  |
|  |  | Liberia | Anglophone | 2467 | 590 |  |  |  |  |
|  |  | Mali | Francophone | 3802 | 974 |  |  |  |  |
|  |  | Nigeria | Anglophone | 11712 | 2926 |  |  |  |  |
|  |  | Niger | Francophone | 4939 | 1480 |  |  |  |  |
|  |  | Senegal | Francophone | 4316 | 1179 |  |  |  |  |
|  |  | Sierra Leone | Anglophone | 4327 | 1208 |  |  |  |  |
| 38 | Kim et al, (2023) | Burkina Faso | Francophone | 1840 | 1840 | Women (average 27yrs) recently delivered with child <6mths | Simple random sampling | Cross-sectional study | High |
| 39 | Koffi et al (2023) | Cote d’Ivoire, | Francophone |  | 980 | Mothers with children aged 0-5mths | Multistage sampling | Cross-sectional study | High |
| 40 | Manyeh et al (2020) | Ghana | Anglophone |  | 1870 | Mothers with the index baby at least 6months registered with DHDSS | Multistage sampling | Cross-sectional study | High |
| 41 | Mohammed et al (2022) | Ghana | Anglophone |  | 3329 | Mothers with children 0-5months | Multistage sampling | Cross-sectional study | High |
| 42 | Morhason-Bello et al (2022) | Nigeria | Anglophone | 11450 |  | 15-49yr old women who have had a childbirth in the past 5 years preceding the surveys. | Simple random sampling | Cross-sectional study | High |
|  |  |  |  | 12349 |  |  |  |  |  |
|  |  |  |  | 16990 |  |  |  |  |  |
|  |  |  |  | 17455 |  |  |  |  |  |
| 43 | Nukpeza et al (2018) | Ghana | Anglophone | 393 | 393 | Mother-infant pairs attending child welfare clinics with children <2yrs | Multistage sampling | Descriptive cross-sectional | High |
| 44 | Oakley et al (2018) | Ghana | Anglophone | Not reported |  | women aged 15–49 with a birth in the 24 months before the survey. | Multi-stage cluster sampling | Cross-sectional study | High |
|  |  | Liberia | Anglophone |  |  |  |  |  |  |
|  |  | Nigeria | Anglophone |  |  |  |  |  |  |
|  |  | Sierra Leone | Anglophone |  |  |  |  |  |  |
|  |  | Benin | Francophone |  |  |  |  |  |  |
|  |  | Burkina Faso | Francophone |  |  |  |  |  |  |
|  |  | Guinea | Francophone |  |  |  |  |  |  |
|  |  | Mali | Francophone |  |  |  |  |  |  |
|  |  | Niger | Francophone |  |  |  |  |  |  |
|  |  | Senegal | Francophone |  |  |  |  |  |  |
| 45 | Oche et al (2011) | Nigeria | Anglophone |  | 179 | Breastfeeding women /stopped breastfeeding within past 2 yrs | Systematic sampling | Descriptive cross-sectional | High |
| 46 | Ogbo et al (2015) | Nigeria | Anglophone | 10225 | 10225 | 15 -49 yrs Women living with youngest living child < 24 months | Stratified two-stage cluster | Cross sectional study | High |
| 47 | Ogbo et al (2017) | Burkina Faso | Francophone | 5710 | 5710 | Mother with children under 24 months | Two-stage sampling | Cross-sectional study | High |
|  |  | Mali | Francophone | 3802 | 3802 |  |  |  |  |
|  |  | Nigeria | Anglophone | 11712 | 11712 |  |  |  |  |
|  |  | Niger | Francophone | 4930 | 4930 |  |  |  |  |
| 48 | Ogunlesi, T. A (2010) | Nigeria | Anglophone | 262 | 262 | Mothers (18-43yrs) of children 1-24 months attending infant welfare clinic | Consecutive sampling | Cross-sectional study | Medium |
| 49 | Okafor et al (2014) | Nigeria | Anglophone | 600 | 600 | Mother (15 - 45yr) with children under 24 months | Multistage sampling | Cross-sectional study | High |
| 50 | Olasinde et al (2021) | Nigeria | Anglophone |  | 271 | Mothers of infants aged less than six months attending immunization clinic | Convenience sampling | Cross-sectional descriptive | High |
| 51 | Olorunsaiye et al (2020) | Senegal | Francophone | 6328 |  | Women 15 to 49yrs with recent live birth within 3 yrs prior to the survey | Two-stage cluster | Cross-sectional study | High |
| 52 | Olorunsaiye et al (2020) | Nigeria | Anglophone | 16077 |  | Women (15-49yr) with live birth within three years prior to the survey | Two-stage cluster | Cross-sectional study | High |
| 53 | Onwuka (2022) | Nigeria | Anglophone | 315 | 315 | Breastfeeding mothers with single babies < 6 months | Convenience sampling | Cross-sectional study | High |
| 54 | Osibogun et al (2018) | Nigeria | Anglophone |  | 200 | Mothers with at least one child | Systematic sampling | Cross-sectional descriptive study | High |
| 55 | Pretorius et al (2021) | Gambia | Anglophone |  | Not reported | children <6 months old who are fed breast milk alone in the past 24 hours. | Not reported | Cross-sectional study | Medium |
|  |  | Ghana | Anglophone |  |  |  |  |  |  |
|  |  | Liberia | Anglophone |  |  |  |  |  |  |
|  |  | Nigeria | Anglophone |  |  |  |  |  |  |
|  |  | Sierra Leone | Anglophone |  |  |  |  |  |  |
|  |  | Benin | Francophone |  |  |  |  |  |  |
|  |  | Burkina Faso | Francophone |  |  |  |  |  |  |
|  |  | Cote d’Ivoire, | Francophone |  |  |  |  |  |  |
|  |  | Guinea Bissau | Francophone |  |  |  |  |  |  |
|  |  | Guinea | Francophone |  |  |  |  |  |  |
|  |  | Mali | Francophone |  |  |  |  |  |  |
|  |  | Niger | Francophone |  |  |  |  |  |  |
|  |  | Senegal | Francophone |  |  |  |  |  |  |
|  |  | Togo | Francophone |  |  |  |  |  |  |
| 56 | Sackey et al (2023) | Ghana | Anglophone | 257 |  | Mothers with children < -24 months | Two-stage cluster | Cross-sectional study | High |
| 57 | Sadoh et al (2011) | Nigeria | Anglophone | 36 | 36 | 25-39 yr old female medical doctors below the level of consultants who had had a baby within the preceding 24 months and who had resumed work. | Convenience sampling | Cross-sectional study | Medium |
| 58 | Senbanjo et al (2014) | Nigeria | Anglophone | 311 | 311 | 12-49 yr old Mothers with children 6-24months attending paediatric clinic | Non-probability sample | Cross-sectional study | High |
| 59 | Setorglo et al (2020) | Ghana | Anglophone | 391 | 391 | Nursing mothers with babies 6-24 months attending clinics | Multistage sampling | Descriptive cross-sectional study | High |
| 60 | Sokan-Adeaga et al 2022 | Nigeria | Anglophone |  | 120 | Mothers aged 15 to 49 years who are currently breastfeeding infants between 0 to 24 months and who are attending postnatal clinic | Non-probability sample | Cross-sectional study | High |
| 61 | Soumah et al (2021) | Guinea | Francophone |  | 851 | Women aged 15 to 49 with their last birth 6 months prior to the data collection | Two-stage sampling | Cross-sectional study | High |
| 62 | Tampah-Naah et al (2013) | Ghana | Anglophone |  | 316 | Mothers of Children 0-5months | Two-stage sample | Cross-sectional study | High |
| 63 | Terefe et al (2023) | Gambia | Anglophone |  | 897 | Mothers with children 0-6months | Stratified, two-stage cluster sampling | Cross-sectional study | High |
| 64 | Teshale et al (2021) | Benin | Francophone | 10309 |  | Women (15-49yr) with child <2yrs | Stratified two-stage cluster | Cross-sectional study | High |
|  |  | Burkina Faso | Francophone | 5739 |  |  |  |  |  |
|  |  | Cote d’Ivoire, | Francophone | 2923 |  |  |  |  |  |
|  |  | Gambia | Anglophone | 1585 |  |  |  |  |  |
|  |  | Ghana | Anglophone | 1153 |  |  |  |  |  |
|  |  | Guinea | Francophone | 1358 |  |  |  |  |  |
|  |  | Liberia | Anglophone | 1266 |  |  |  |  |  |
|  |  | Mali | Francophone | 3850 |  |  |  |  |  |
|  |  | Nigeria | Anglophone | 4801 |  |  |  |  |  |
|  |  | Niger | Francophone | 2351 |  |  |  |  |  |
|  |  | Senegal | Francophone | 2340 |  |  |  |  |  |
|  |  | Sierra Leone | Anglophone | 2117 |  |  |  |  |  |
|  |  | Togo | Francophone | 1312 |  |  |  |  |  |
| 65 | Uchendu et al (2009) | Nigeria | Anglophone |  | 184 | Women with children > 6 months attending a paedics clinic | Systematic sampling | Cross-sectional study | Medium |
| 66 | Wan et al (2023) | Gambia | Anglophone | 7581 |  | Mothers (15-49yr) who delivered at a healthcare facility who have had children within 5yrs of survey | Stratified two-stage cluster | Cross-sectional study | High |
|  |  | Liberia | Anglophone | 5235 |  |  |  |  |  |
|  |  | Nigeria | Anglophone | 33711 |  |  |  |  |  |
|  |  | Sierra Leone | Anglophone | 9779 |  |  |  |  |  |
|  |  | Benin | Francophone | 13446 |  |  |  |  |  |
|  |  | Guinea | Francophone | 7890 |  |  |  |  |  |
|  |  | Mali | Francophone | 10286 |  |  |  |  |  |
|  |  | Senegal | Francophone | 5600 |  |  |  |  |  |
| 67 | Yakubu et al (2023) | Nigeria | Anglophone |  | 230 | Nursing mothers (20 -50yrs) with babies less than 2 yrs old | Simple random sampling | Descriptive and cross-sectional | High |
| 68 | Yalçin et al (2016) | Ghana | Anglophone |  | 317 | Women (15-49yr) with infants <6months | Two-stage sample | Cross-sectional study | High |
|  |  | Liberia | Anglophone |  | 497 |  |  |  |  |
|  |  | Nigeria | Anglophone |  | 2833 |  |  |  |  |
|  |  | Sierra Leone | Anglophone |  | 590 |  |  |  |  |
|  |  | Benin | Francophone |  | 1118 |  |  |  |  |
|  |  | Burkina Faso | Francophone |  | 1452 |  |  |  |  |
|  |  | Cote d’Ivoire, | Francophone |  | 770 |  |  |  |  |
|  |  | Guinea | Francophone |  | 714 |  |  |  |  |
|  |  | Mali | Francophone |  | 1418 |  |  |  |  |
|  |  | Niger | Francophone |  | 1299 |  |  |  |  |
|  |  | Senegal | Francophone |  | 1318 |  |  |  |  |
| 69 | Yeboah et al (2019) | Ghana | Anglophone |  | 160 | Lactating mothers with child 6 - 24 months | Two-stage sample | Cross-sectional study | High |
